# Investigation of Radiotracer Metabolic Stability In Vitro with CYP-Overexpressing Hepatoma Cell Lines

**DOI:** 10.3390/cells11152447

**Published:** 2022-08-07

**Authors:** Sandy Lemm, Susanne Köhler, Robert Wodtke, Friedrich Jung, Jan-Heiner Küpper, Jens Pietzsch, Markus Laube

**Affiliations:** 1 Helmholtz-Zentrum Dresden-Rossendorf, Institute of Radiopharmaceutical Cancer Research, Bautzner Landstrasse 400, 01328 Dresden, Germany; 2 Faculty of Chemistry and Food Chemistry, School of Science, Technische Universität Dresden, Mommsenstrasse 4, 01062 Dresden, Germany; 3 Institute of Biotechnology, Brandenburg University of Technology Cottbus-Senftenberg, Universitätsplatz 1, 01968 Senftenberg, Germany

**Keywords:** cytochrome P450 enzymes, cyclooxygenase-2 inhibitors (coxibs), drug testing models, HepG2 cells, liver microsome assay, mass spectrometry, metabolic radiotracer stability assay, model reliability, radio thin-layer-chromatography (radio-TLC), pharmacokinetics

## Abstract

The characterization of novel radiotracers toward their metabolic stability is an essential part of their development. While in vitro methods such as liver microsome assays or ex vivo blood or tissue samples provide information on overall stability, little or no information is obtained on cytochrome P450 (CYP) enzyme and isoform-specific contribution to the metabolic fate of individual radiotracers. Herein, we investigated recently established CYP-overexpressing hepatoblastoma cell lines (HepG2) for their suitability to study the metabolic stability of radiotracers in general and to gain insight into CYP isoform specificity. Wildtype HepG2 and CYP1A2-, CYP2C19-, and CYP3A4-overexpressing HepG2 cells were incubated with radiotracers, and metabolic turnover was analyzed. The optimized protocol, covering cell seeding in 96-well plates and analysis of supernatant by radio thin-layer-chromatography for higher throughput, was transferred to the evaluation of three ^18^F-labeled celecoxib-derived cyclooxygenase-2 inhibitors (coxibs). These investigations revealed time-dependent degradation of the intact radiotracers, as well as CYP isoform- and substrate-specific differences in their metabolic profiles. HepG2 CYP2C19 proved to be the cell line showing the highest metabolic turnover for each radiotracer studied here. Comparison with human and murine liver microsome assays showed good agreement with the human metabolite profile obtained by the HepG2 cell lines. Therefore, CYP-overexpressing HepG2 cells provide a good complement for assessing the metabolic stability of radiotracers and allow the analysis of the CYP isoform-specific contribution to the overall radiotracer metabolism.

## 1. Introduction

There is an ongoing need for the development of specific radiopharmaceuticals that will most prominently and specifically bind to malignant tissue to obtain high contrast between healthy and pathologic tissue, as well as functional information about pathophysiological processes in vivo. Currently, there exist a variety of radiotracers ranging from small-molecule compounds (e.g., carbohydrates, amino acids, or steroids) to more complex molecules of higher molecular weight (peptides, proteins, or oligonucleotides) [1]. Usually, small-molecule-based radiotracers are rapidly distributed within and cleared from the organism [2]. However, radiometabolites that arise from different metabolic pathways can be troublesome due to their different biodistribution and potential impact on tracing the original target compound. In this context, radiodefluorination represents a common metabolic transformation for ^18^F-labeled radiotracers that can be problematic. This process is known to take place during phase I of drug metabolism, mainly mediated through the action of cytochrome P450 enzymes [3,4,5]. The resulting [^18^F]fluoride accumulates then in the skeleton [1].

In general, the metabolism or biotransformation aims at increasing the hydrophilicity of a molecule to enhance its renal and/or hepatobiliary excretion, which is achieved via mainly three phases of chemical conversion: (I) functionalization, (II) conjugation, and (III) excretion. These processes take place mostly in the liver and kidney with the family of cytochrome P450 (CYP) enzymes being mainly responsible for the oxidative biotransformation of xenobiotics. Among the different CYP isoforms, CYP3A4 is the most abundant and prominent isoform; it metabolizes >50% [6,7] of all known drugs that undergo phase I conversion. Significant expression of CYPs can also be found in extrahepatic tissue, such as the respiratory or gastrointestinal tract, which are at the same portals for xenobiotics to enter the body [8]. Most radiotracers are intravenously injected, which limits the first-pass effect by circumventing the gastrointestinal absorption.

Common preclinical practice to investigate metabolic stability of radiotracers involves in vitro stability assays with liver microsomes and plasma, as well as blood sampling after intravenous (i.v.) injection of the radiotracers in mice or rats [9]. For preclinical screening of novel compounds, in vitro test systems are of particular importance. In general, in vitro test systems can follow two different approaches: the use of subcellular fractions (e.g., microsomes or reconstituted recombinant enzymes) or the use of competent cellular models [10,11,12,13]. While primary human hepatocytes are generally referred to as the gold standard, different alternatives have been explored to improve the limitations of this cellular model (i.e., phenotypic instability, restricted accessibility, and limitations to generate reproducible data) [14]. Although the in vitro metabolite profile of a drug generally reflects those of in vivo systems, in vitro results do not always correlate well to the in vivo performance of radiotracers [15]. HepG2 cells show many liver-specific functions, but their drawback is the lack of functional expression of almost all relevant human liver phase I (CYP450) and phase II enzymes [16,17]. Over the past years, different HepG2 cell lines with overexpression of CYP1A2 [18], CYP2C9 [19] CYP2C19 [19,20], CYP2D6 [19], CYP3A4 [19,21], and POR (CYP oxidoreductase) [19] were generated to overcome the lack of functional CYP expression. A stable overexpression of CYP1A2 [18], CYP2C19 [20], and CYP3A4 [21] was enabled through lentiviral transfection and proved to be a better reflection of the in vivo situation than the parental HepG2 cell line [20]. These cell lines can be used to evaluate the role of specific CYP isoforms in the metabolism of certain drugs and to assess the metabolism-associated toxicity of drugs [22]. Moreover, the suitability of these cell lines to predict the metabolic fate of pharmaceuticals such as cyclophosphamide [23], testosterone [21], tolbutamide [20], and phenacetin [18] was already proven. Therefore, we hypothesized that the stability of radiotracers could also be investigated with these cell lines.

For cyclooxygenase (COX), two different isoforms are known; COX-1 is constituently expressed, while COX-2 is absent in healthy tissues with the exception of heart, kidney, and brain, but is inducible under inflammatory conditions. Radiotracers that target COX-2 are, therefore, under investigation to image inflammatory processes and tumors in vivo [24,25,26,27]. On the basis of our interest in radiolabeled COX-2 inhibitors and our previous work covering the liver microsomal stability of fluorine-18-labeled celecoxib derivatives, compounds **[^18^F]1–3** [28], subsequently also referred to as ^18^F-labeled coxibs, were chosen for this study. Compound **[^18^F]1** (3-([^18^F]fluoromethyl)-1-[4-(methylsulfonyl)phenyl]-5-(*p*-tolyl)-1*H*-pyrazole) and the respective deuterated (**[^18^F]2**) and ethylated (**[^18^F]3**) derivatives belong to the class of COX-2 inhibitors and were investigated toward their metabolic stability in vivo and in vitro with the murine liver microsome (MLM) assay. Both in vivo and in vitro analysis revealed an increase in metabolic stability through deuteration [28]. The lead compound celecoxib is mainly metabolized by CYP2C9 and CYP3A4, while the contribution of other isoforms is not yet reported [29].

Herein we present a methodology to investigate the metabolic stability of radiotracers by the use of CYP-overexpressing HepG2 cell lines. The obtained data highlight CYP and substrate specificities for the metabolic stability of three COX-2-targeting radiotracers. Moreover, the established protocol using radio-TLC for analyzing the metabolic transformations allows for a simple sample preparation. Overall, our cell-based approach adds a further attractive method to the toolbox of in vitro methods for assessing the metabolic stability of radiotracers.

## 2. Materials and Methods

### 2.1. General

All commercial reagents and solvents were used without further purification. The utilized radiotracers are named as follows according to IUPAC nomenclature: **[^18^F]1** (3-([^18^F]fluoromethyl)-1-[4-(methylsulfonyl)phenyl]-5-(*p*-tolyl)-1*H*-pyrazole), **[^18^F]2** (3-([D_2_, ^18^F]fluoromethyl)-1-[4-(methylsulfonyl)phenyl]-5-(*p*-tolyl)-1*H*-pyrazole), and **[^18^F]3** (3-([^18^F]fluoroethyl)-1-[4-(methylsulfonyl)phenyl]-5-(*p*-tolyl)-1*H*-pyrazole). Radiotracers were prepared following the previously described route [28] with the following minor modifications: (i) reactions were carried out using manual radiosyntheses under application of a microliter scale radiofluorination approach [30]; (ii) radiofluorination toward **[^18^F]1** and **[^18^F]2** was carried out at 90 °C in dry acetonitrile (ACN), while **[^18^F]3** was obtained after reaction at 130 °C in DMSO; (iii) semipreparative HPLC purification (Nucleosil 100-7 C18, 250 × 16 mm, 100 Å, Macherey Nagel; 5 mL/min isocratic flow 0.1% TFA in ACN/0.1% TFA in water: 50/50, Shimadzu prominence modular HPLC system) allowed isolation of the respective radiotracers after ~20–22 min separation time in usually two fractions, one containing a lower content of chemical impurity that was finally selected for further processing by solid phase extraction and formulation in EtOH for cell experiments. **[^18^F]1–3** were isolated in radiochemical yields of 30–70% (after HPLC based on [^18^F]fluoride after QMA elution) with radiochemical purity exceeding 99% according to HPLC, chemical purity between 35% and 65%, and a molar activity in the range of 20–150 GBq/µmol at the end of synthesis.

### 2.2. Liquid Chromatography (LC)

Analytical HPLC was performed with the following systems and corresponding elution profiles: (System 1) Shimadzu Nexera X2 UHPLC system (Kyoto, Japan; degasser DGU-20A3R and DGU-20A5R, pump LC-30AD, autosampler SIL-30AC, column oven CTO-20AC with two column switching valves FCV-14AH, diode array detector SPD-M30A, γ detector Gabi Star (Raytest, Straubenhardt, Germany), communication bus module CBM-20A); eluent: (A) ACN, (B) 0.1% trifluoroacetic acid in H_2_O; (a) UHPLC column C_18_ Kinetex (Phenomenex 50 × 2.1 mm, 1.7 µm, 100 Å); flow rate 0.5 mL/min; 25–75% gradient (t_0 min_ 25/75—t_0.3 min_ 25/75—t_4.0 min_ 75/25—t_4.5 min_ 95/5—t_5.5 min_ 95/5—t_6.0 min_ 25/75—t_7.5 min_ 25/75); 50% isocratic elution (t_0 min_ 50/50—t_10 min_ 55/45—t_11 min_ 95/5—t_18 min_ 95/5—t_19 min_ 50/50—t_22 min_ 50/50), (b) HPLC column C_18_ Luna (Phenomenex, 5 µm, 250 × 4.6 mm); flow rate 1.0 mL/min; 25–75% gradient (t_0 min_ 25/75—t_3 min_ 25/75—t_28 min_ 75/25—t_29 min_ 95/5—t_34 min_ 95/5—t_35 min_ 25/75—t_40 min_ 25/75). (System 2) Waters UPLC I-Class (Milford, MA, USA; binary gradient pump BSM, autosampler FTN, column manager CM, and diode array detector PDAeλ coupled to Waters Xevo TQ-S), column Aquity UPLC^®^ BEH C_18_ column (waters, 100 × 2.1 mm, 1.7 µm, 130 Å), eluent: (A) 0.1% acetic acid in ACN/MeOH 1/1, (B) 0.1% acetic acid in H_2_O; flow rate 0.4 mL/min, 25–75% gradient (t_0 min_ 25/75—t_0.5 min_ 25/75—t_5.5 min_ 75/25—t_6.0 min_ 95/5—t_7.0 min_ 95/5—t_8.0 min_ 25/75—t_8.5 min_ 25/75); 45% isocratic elution (t_0 min_ 45/55—t_5.5 min_ 45/55—t_6 min_ 95/5—t_7 min_ 95/5—t_7.5 min_ 45/55—t_8.5 min_ 45/55); 50% isocratic elution (t_0 min_ 50/50—t_5.5 min_ 50/50—t_6 min_ 95/5—t_7 min_ 95/5—t_8.0 min_ 50/50—t_8.5 min_ 50/50); 55% isocratic elution (t_0 min_ 55/45—t_3 min_ 55/45—t_3.2 min_ 95/5—t_3.8 min_ 95/5—t_4 min_ 55/45—t_4.2 min_ 55/45). The products were monitored at λ = 254 nm and, in the case of radio-HPLC, additionally using the γ detector. Prior injection sampled needed to be precipitated. Therefore, the supernatant was mixed with ACN (1/4, *v*/*v*) and centrifuged at 14,000 rpm (18,800–28,000× *g*) for 5 min at 4 °C.

### 2.3. Mass Spectrometry (MS)

Low-resolution mass spectra were obtained using positive electrospray ionization (ESI+) on a Waters Xevo TQ-S coupled to a UPLC I-Class (System 2) operated with MassLynx (version 4.1). If needed, results were quantified using the TargetLynx software (version 4.1). For this, the multiple reaction monitoring (MRM) mode was utilized, which tracks specific ion transitions under optimized fragmentation conditions (cone and collision voltage). The setup for the Xevo TQ-S MS detector involved 150 °C source temperature, 500 °C desolvation temperature, 150 L/h cone gas, and 1000 L/h desolvation gas (argon). Specific parameters for used compounds are listed in the respective sections below. The detected signals for each specific fragmentation were integrated with the TargetLynx software.

### 2.4. Radio Thin-Layer Chromatography (Radio-TLC)

Radio thin-layer chromatography (radio-TLC) was carried out on reversed-phase TLC plates (ALUGRAM RP-18W/UV_254_ from Macherey-Nagel, Düren, Germany) with a mobile phase of 45% ACN in water containing 0.1% trifluoroacetic acid. The samples (2 µL) were spotted onto the TLC plate. The line of origin and the front line were labeled with 1/5 of the sample activity concentration. To track the R_f_ value of the intact radiotracer, each TLC was spotted with the intact tracer as reference in the middle. Detection of the activity signals was achieved after 10–30 min development on radioluminographic imaging plates (FUJIFILM, Tokyo, Japan) and subsequent readout with Fuji BAS 5000^®^ or Amersham™ Typhoon™ from GE Healthcare (Uppsala, Sweden). The resulting chromatographic profile was analyzed and integrated with the help of AIDA Image Analysis Software (version 5.1). For data depiction, the fraction of each species (intact radiotracer, metabolite, [^18^F]fluoride) was plotted.

### 2.5. Cell Culture

CYP-overexpressing human hepatocellular carcinoma cells (HepG2) were previously generated on the basis of subcloning human CYP1A2 [18], CYP2C19 [20], and CYP3A4 [21] cDNA into a lentiviral expression vector. The cDNA expression in the resulting vector was controlled by the human cytomegalovirus (CMV) promoter. The lentivirus expression vectors were used to produce recombinant lentivirus particles according to standard methods to infect HepG2 cells. Infected cells were selected with 3 µg/mL blasticidin for CYP2C19 and CYP3A4 and 500 µg/mL zeocin for CYP1A2 overexpression 48 h after infection. Within 3–4 weeks, antibiotic-resistant cell colonies were picked to obtain single cell-derived HepG2 clones. For more detailed information on the generation procedure of CYP-overexpressing HepG2 cells, see Steinbrecht et al. [18].

HepG2 cells were cultured in DMEM GlutaMax (Gibco, Paisley, UK) with 10% fetal calf serum (FCS) and with selective antibiotics (penicillin/streptomycin for wildtype, Zeocin (InvivoGen, San Diego, CA, USA) for CYP1A2, and Blasticidin (Gibco, Carlsbad, CA, USA) for CYP2C19 and CYP3A4 cultures). As a negative control, cultures of CHO cultivated in Ham’s F-12 medium (Gibco) with 10% FCS and 2 mM glutamine were added for the radiotracer experiments. Cells were handled under a laminar flow hood (Thermo Scientific^TM^ Safe 2020, Langenselbold, Germany) and were split once or twice a week by trypsinization. Cell cultures were stored horizontally in a humidified incubator (Heracell240i) at 37 °C and 5% CO_2_. During the time of experimental work, the utilized cell lines were regularly checked for mycoplasma. Cells were seeded in duplicates into a 96-well plate (CellStar^®^) with a density of 3 × 10^5^ cells/well and cultured until the next day at 37 °C and 5% CO_2_. Cell density was determined with a cell counter (CASY^®^ Model TT from Schärfe System, Reutlingen, Germany). Before the addition of substrates, cells were washed twice with Krebs–Henseleit buffer (KHB). The HEPES-supplemented Krebs–Henseleit buffer (pH 7.4, KHB) was freshly prepared using HEPES (5.95 g/L), d-glucose (2.0 g/L), magnesium sulfate (0.141 g/L), potassium dihydrogenphosphate (0.16 g/L), potassium chloride (0.35 g/L), sodium chloride (6.9 g/L), calcium chloride dihydrate (0.373 g/L), and sodium bicarbonate (2.1 g/L), followed by adjustment to pH 7.4 with NaOH. The utilized substances within this study were neither found (see Supplementary Materials, Figure S14) nor reported [31,32,33,34,35] to be cytotoxic within the utilized concentration range and incubation duration.

Depending on the specific cell line, cells were utilized in the range of P17–22 (CHO), P6–17 (HepG2 wildtype), P19–31 (HepG2 CYP1A2), P33–42 (HepG2 CYP2C19), and P29–33 (HepG2 CYP3A4).

### 2.6. Radiotracer Incubation

Incubations were conducted for up to 2 h in a 96-well plate in KHB with a total incubation volume of 50 µL and an ethanol concentration of 0.4%. For the incubation with radiotracers, an activity of 100–200 kBq/well at the start of the experiment was used.

Incubations with radiotracer compounds were conducted, on the one hand, with radioactive ^18^F-labeled compounds alone (time dependency) and, on the other hand, in the presence of 50 µM nonradioactive reference (carrier addition). For the investigation of a time-dependent metabolite formation, 2 µL aliquots were withdrawn from the supernatant, directly spotted onto the TLC plate, and analyzed as described above. The same procedure was performed for carrier-added samples.

UPLC–MS analyses were performed to verify the degradation behavior of the test compounds and to gain insights into the identity of the formed metabolites. Using radiotracer incubation samples, 40 µL aliquots of the carrier-added incubations were withdrawn followed by addition of 160 µL of ACN and centrifugation (14,000 rpm (18,800–20,800× *g*), 4 °C, 5 min). Samples were frozen at −20 °C, and activity was allowed to decay before further mass-spectrometric analyses. Additionally, incubations with the nonradioactive references were carried out with 100 nM or 50 µM (according to the carrier-added samples) and analyzed. UPLC–MS/MS was performed using System 2 (55% isocratic elution for quantification of intact compound, or 25–75% gradient for nonradioactive metabolite analysis). The MRM mode was optimized with a solution of respective reference compounds diluted in eluent. The following specific parameters were used: compound **1** was monitored at transitions for [M + H]^+^ 345.18 > 231.11 (38 V collision energy), 345.18 > 245.40 (38 V collision energy), and 345.18 > 266.20 (28 V collision energy) at 4 V cone voltage; compound **2** was monitored at transitions for [M + H]^+^ 347.15 > 248.05 (26 V collision energy) and 347.15 > 268.14 (28 V collision energy) at 70 V cone voltage; compound **3** was monitored at transitions for [M + H]^+^ 359.08 > 206.37 (40 V collision energy), 359.08 > 260.00 (30V collision energy), and 359.08 > 280.14 (30 V collision energy) at 42 V cone voltage.

### 2.7. Testosterone Incubation

Testosterone (SIGMA-ALDRICH, Taufkirchen, Germany) was dissolved in pure ethanol. Control incubations with testosterone were carried out for 2 h with 2.5 µM testosterone, 0.05% ethanol, and a total volume of 150 µL in the cavities of a 96-well plate. The supernatant was prepared and analyzed by UPLC–MS/MS (System 2; 45% isocratic elution). Testosterone was monitored at transitions for [M + H]^+^ 289.24 > 97.08 and 289.24 > 109.05 with cone voltage 52 V and collision energy 20 V, while the CYP-mediated metabolite androstenedione was monitored at transitions for [M + H]^+^ 287.22 > 97.08 (collision energy 18 V) and 287.22 > 109.05 (collision energy 20 V) with a cone voltage of 50 V each.

### 2.8. Microsome Assay

The stability of **[^18^F]1** toward liver microsomes was analyzed under oxidative conditions with MLMs (MSMCP, Lot: MS046-C) and HLMs (HMMCPL; Lot: PL050E-B) according to a previously described procedure [28,36]. In brief, 12.5 µL of the respective microsomes (final 1 mg/mL) were mixed with 110.4 µL of PBS, followed by the addition of 100 µL of **[^18^F]1** (with 5 MBq and 1% ethanol; final 0.4% ethanol) and 2.1 µL of reference compound (final concentration 50 µM **1**), or an equivalent of DMSO. The resulting mixture was mixed at 37 °C for 5 min, and the reaction was then initiated by the addition of 25 µL of NADPH (final 2 mM). After 1 h incubation, the resulting samples were mixed with ice-cold ACN (1/4, *v*/*v*) and vortexed for 30 s, placed on ice for 4 min, and finally centrifuged at 13,200 rpm for 5 min at 4 °C. The supernatant was separated and used for further analysis by radio-HPLC (6–10 µL injection volume; System 1b with 25–75% gradient elution) and radio-TLC analysis (2 µL applied on TLC, analysis as described above).

## 3. Results and Discussion

### 3.1. Method Development

Investigations of the in vitro metabolism of ^18^F-labeled radiotracers using liver microsomes is commonly performed by incubating the radiotracer with pooled liver microsomes at a concentration of approximately 1 mg/mL in the presence of NADPH. Analysis can then be achieved by radio-HPLC or radio-TLC to detect formation of ^18^F-containing metabolites or free [^18^F]fluoride. In our previous work, we investigated the oxidative transformations of **[^18^F]1–3** using murine liver microsomes in detail. All three compounds underwent conversion to more hydrophilic ^18^F-bearing metabolites (herein named **M1**, **M2**, and **M3**, respectively) and showed ^18^F-defluorination. These data and the applied analytical methods served as the basis for the investigations using the HepG2 cell lines.

**Figure 1:**
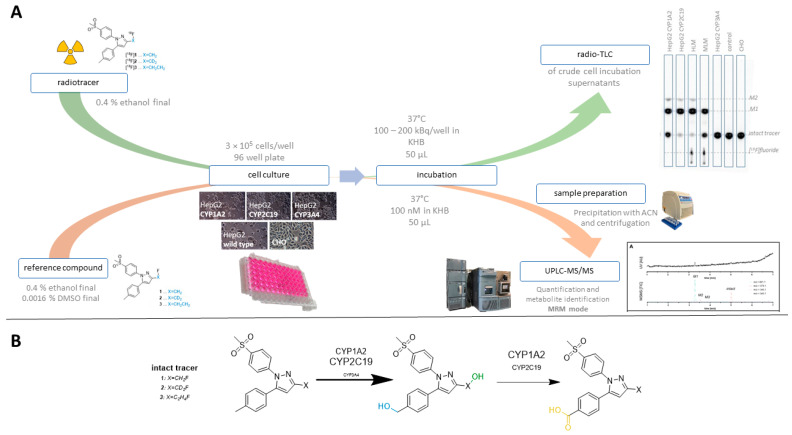
Established workflow for investigating the metabolism of radiotracers. (**A**) Workflow for incubations with radiotracers (green arrow) and for nonradioactive reference compounds (orange arrow). (**B**) Sites for oxidative metabolism in **[^18^F]1–3** [28,29].

Initially, CYP-overexpressing HepG2 and, as controls, HepG2 wildtype, as well as CHO (Chinese hamster ovary) cells, were incubated as an adhered monolayer in a 96-well plate with a relatively high activity concentration of **[^18^F]1** (25 MBq/mL; workflow according to Figure 1A, upper panel), and the supernatant and cell lysates were investigated by radio-TLC. For HepG2 CYP2C19 and CYP1A2 cell lines, the formation of radiometabolites was observed in both incubation supernatant and cell lysates, while, for HepG2 wildtype, HepG2 CYP3A4, and CHO cells, mainly intact radiotracer was found in both fractions (see Supplementary Materials; Figure S1). The activity distribution between supernatant and cells was investigated for all utilized cell lines at the end of the incubation period (2 h). As a result (Table 1), the main fraction of activity was found in the supernatant (64–91%), while only a negligible to low amount of activity was measured in cell lysates (1–16%). Residual activity was found in the washing solution (9–13%) or could be attributed to losses on plastics such as tips or vials. Accordingly, it was concluded that, for this class of radiotracers, the metabolic turnover could principally be followed using an adhered monolayer culture of HepG2 cells, and that the analysis of the supernatant was representative for the metabolic transformations.

Analysis of metabolites can principally be performed by radio-HPLC or radio-TLC, both having their own advantages and drawbacks. While radio-TLC can be regarded as more sensitive with regard to the detectable activity concentration and offers higher throughput in a short time, radio-HPLC offers, amongst others, a higher chromatographic resolution. Experiments with **[^18^F]1** showed that the formed metabolites within this study could be well resolved by radio-TLC and radio-HPLC, but lower activity concentrations (100–200 kBq/well in a 96-well plate) were reliably detected only by radio-TLC. As a further advantage, we found that the supernatant could be directly analyzed by radio-TLC without further work-up to precipitate proteins as usually required for radio-HPLC.

Different culturing conditions were tested including the well format (24- versus 96-well plates—see Supplementary Materials, Figure S3), activity concentration (15.7 MBq/mL versus 1.5 MBq/mL—see Supplementary Materials, Figure S2), and duration (2 versus 4 h—see Supplementary Materials, Figure S2). The use of 96-well plates with an incubation volume of 50 µL and an incubation period of max 2 h turned out to be most favorable considering the detectability of potential metabolites and the short half-life of fluorine-18 (t_½_ 109.77 min). Each setup revealed the presence of a metabolite with increased hydrophilicity (**M1**), while a second metabolite with even higher hydrophilicity (**M2**) was more present in incubations with lower activity concentration (see Supplementary Materials, Figure S2). Accordingly, a lower activity concentration was chosen for the radiotracer incubations, and radio-TLC was used for analyzing the metabolic transformations. However, we recommend that these parameters should be tested for each substance class as part of the specific optimization process.

Due to the known interferences of organic cosolvents on the enzymatic activity of recombinant CYP enzymes [37,38], the influence of different concentrations (0.5%, 1.5%, and 3%) of ethanol, ACN and DMSO, which were used for the radiosyntheses or as solvents for the reference compounds, on the cellular CYP activity was tested (see Supplementary Materials, Figure S4). At the lowest solvent concentration of 0.5%, complete turnover of **[^18^F]1** to the two polar metabolites **M1** and **M2** and [^18^F]fluoride was observed for ethanol, ACN, or DMSO. None of the analyzed solvent concentrations yielded a change in the metabolic profile (no other metabolites were observable) or a complete inhibition of metabolic turnover for **[^18^F]1**. Acetonitrile had no impact on the distribution within the metabolite profile, while, for ethanol and DMSO, an increase in **M1** and a decrease in **M2** were observed in the presence of increasing solvents concentrations. Exemplarily, an increase in **M1** from 46% to 59% was observed for 0.5% to 3.0% of ethanol in HepG2 CYP1A2 cells. Notably, also at the highest solvent concentration where isolated enzymes were already markedly or completely inhibited [37,38], the metabolic turnover in the cell-based assay was still detected. To keep the influence of solvent minimal, a standardized solvent concentration of 0.4% ethanol for the radiotracer incubations was applied.

In order to establish representative negative controls, we utilized incubations of the respective radiotracer in buffer only (control) and incubations using an adhered monolayer of Chinese hamster ovarian (CHO) cells in a comparable setting. For both, no metabolic turnover of **[^18^F]1** was observed.

**Figure 2:**
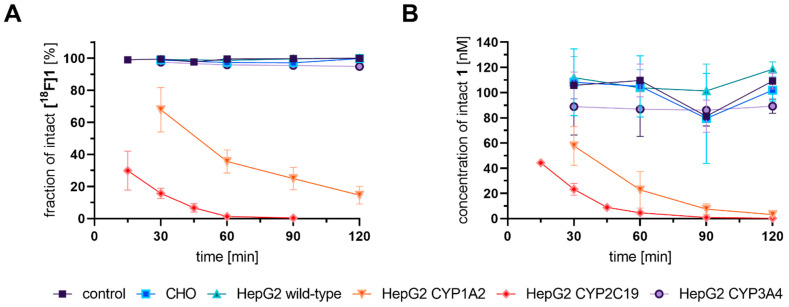
Time-dependent degradation of **[^18^F]1** measured with radio-TLC (**A**) and **1** followed with LC–MS/MS (**B**) upon incubation with CYP-overexpressing cell lines. (**A**) Plot of fraction of intact **[^18^F]1** in the supernatant of indicated cell lines after 30, 60, 90, and 120 min as analyzed with radio-TLC. For HepG2 CYP2C19, additional timepoints at 15 min and 45 min were analyzed. Incubations were performed with 2–4 MBq/mL of **[^18^F]1** corresponding to a concentration range of 28–187 nM. Data shown are mean values (±SD) with n = 4. (**B**) Plot of intact **1** in the supernatant of indicated cell lines at the same timepoints as stated in (A). Incubations were performed with 100 nM **1**. Analysis in the supernatant with prior precipitation of proteins was performed by UPLC–MS/MS (System 2, 55% isocratic elution) and MRM following indicated ion transitions. Data shown are mean values (±SD) with *n* = 4.

As a further control, we aimed to verify degradation of our reference compounds in a nonradioactive setting that is comparable to traditional HPLC-based methods and at concentrations comparable to the radiotracer experiments (Figure 1—orange path). With regard to our optimized procedure and molar activities (A_m_) in the range of 20–160 GBq/µmol (corresponding to a utilized concentration of 2.3–3.7 MBq/mL at start of experiment), the applied concentrations of the ^18^F-labeled radiotracers were in the range of 29–187 nM. While these concentrations are near the detection limit of UV-based HPLC detectors, they are sufficiently high for analysis by UPLC–MS/MS detection in multiple reaction monitoring (MRM) mode. In contrast to the quantitative detection of different ^18^F-bearing analytes by the measurement of the activity, the quantitative detection via UPLC–MS/MS required a standard of the respective analyte such that only reference compounds could be analyzed within this study. For this reason, the degradation of the respective intact reference compound was analyzed at a concentration of 100 nM, which corresponded well to the applied radiotracer concentration. As shown in Figure 2, the trends from the radioactive setup could be confirmed by UPLC–MS/MS; HepG2 CYP1A2 and CYP2C19 showed tracer degradation, while results for remaining cell lines were comparable to the negative control. This good comparability between radioactive and nonradioactive analysis was observed for all three investigated tracers (depicted in detail in Supplementary Materials, Figure S7). On this basis, the approach of analyzing nonradioactive reference compounds with LC–MS prior to more elaborated radiolabeling can be suggested as a useful preliminary test.

Because radiotracer candidates are produced in batches and the experiments are repeated over the course of days to weeks, a control setting was established that allowed assessment of the overall metabolic activity of the cells. Principally, CYP overexpressing HepG2 cell lines were shown to exhibit a dedicated substrate specificity. However, we found that the conversion of testosterone to androstenedione, which is principally a CYP P450-mediated C17 oxidation [39], occurred in all four cell lines and was an appropriate and more general marker for the functionality of the cell lines. For the analysis, LC–MS/MS in MRM mode was performed following the degradation of testosterone and the formation of androstenedione (see Supplementary Materials, Figure S6). Accordingly, functional and well-cultured HepG2 cells (3 × 10^5^ cells/well in 96-well plate) consumed more than 50% of an applied testosterone concentration of 2.5 µM within 2 h, accompanied by the formation of more than 1 µM androstenedione.

Final culture conditions and parameters were set as follows; cells were seeded in duplicates into a 96-well plate with a density of 3 × 10^5^ cells/well and cultured until the next day. Corresponding substrates were diluted in KHB (and, if necessary, in additional solvents) to the desired concentration. After incubation, the supernatant was prepared for the corresponding analysis by radio-HPLC/TLC or UPLC–MS/MS. Control incubations with testosterone were simultaneously performed, and results from tracer incubations were evaluated only when the obtained data from the testosterone incubations proved functional CYP activity and cell viability (Figure 1).

### 3.2. Comparison with Microsome Assay

**Figure 3:**
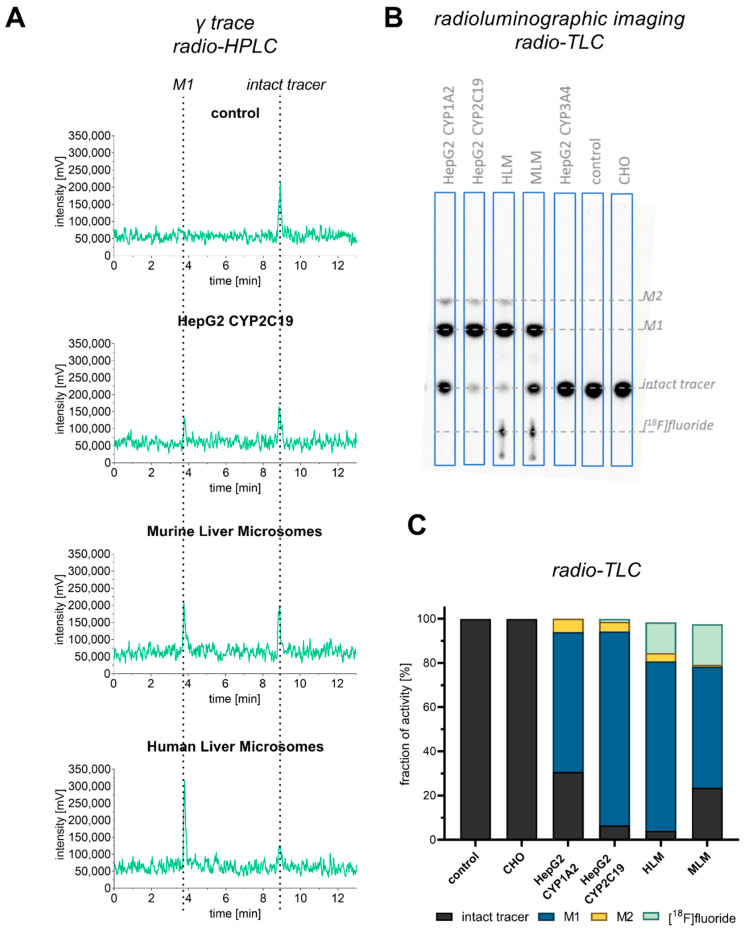
Comparison of cell-based and microsome-based **[^18^F]1** metabolism after 1 h incubation. For the microsome assay, 38 MBq/mL of **[^18^F]1** corresponding to 931 nM was applied, while, for the cell-based assay, 24 MBq/mL corresponding to 460 nM was used. (**A**) Radio-HPLC chromatogram of selected supernatant, which was precipitated and analyzed with System 1a, 50% isocratic elution. Compounds were detected with γ-detector. Peaks (activity) were detected in the γ trace at 3.8 min for **M1** and 8.9 min for the intact tracer. (**B**) Representative radio-TLC scan with assignment to the **^18^**F-bearing species. (**C**) Fraction of activity distribution in supernatant after cell or microsome incubation as obtained from radio-TLC analysis, *n* = 1. The intact tracer (black) gets metabolized to hydroxylated metabolite M1 (blue) and carboxylated metabolite M2 (yellow) under defluorination (mint).

Next, we sought to compare the results obtained with the CYP-overexpressing HepG2 cell lines with liver microsome assays as gold standard. For the comparison, the metabolic stability of **[^18^F]1** was simultaneously investigated with HepG2-overexpressing cell lines, human liver microsomes (HLMs), and murine liver microsomes (MLMs). The obtained data are collectively represented in Figure 3 and in Supplementary Materials, Figure S5. Radio-HPLC confirmed the same retention times of intact tracer (8.7 min) and the radiometabolite **M1** (3.6 min) (Figure 3A).

As also obtained from radio-TLC analyses, the metabolic profiles and corresponding percentual distribution of radioactive labeled compounds (Figure 3B,C) of the HLM and HepG2 CYP2C19 incubation were comparable with the exception of an increased amount of [^18^F]fluoride formed in microsome samples. Both samples contained intact tracer, one major metabolite (**M1**), and notably, an additional second metabolite (**M2**) with similar intensities. Interestingly, the second metabolite (**M2**) was observed in incubations with human but not murine liver microsomes, which indicates species differences in the metabolic transformations of **[^18^F]1**. The radio-TLC scans depicted in Figure 3B clearly confirmed that the intact tracer and the formed metabolites had the same retention times regardless of the utilized assay method. With respect to the carrier-added (CA) incubation (see Supplementary Materials, Figure S5) differences in the extent of metabolism between HLM and HepG2 CYP2C19 were detected. The amount of intact tracer within HepG2 CYP2C19 incubation increased 10-fold (6.6% without carrier addition, 67.2% with CA) while the HLM intact tracer amount increased only sevenfold (4.2% without carrier addition, 30.3% with CA).

Due to similar metabolite profiles of HepG2 CYP2C19 and HLMs, CYP2C19 could be assumed to modulate the maximal turnover of **[^18^F]1**, since HLMs represent the in vivo expression of different CYP isoforms in the most applicable manner within available in vitro assays. However, since no information of the precise protein expression was investigated within this study, a quantitative comparison between the three CYP isoforms is not possible. For such aims an additional assay with isolated enzymes is recommended. Furthermore, the CYP-overexpressing cell lines have proven the advantage of originating from human tissue. A simultaneous comparison with HLMs and MLMs (Figure 3) revealed that murine liver microsome incubation did not lead to the second metabolite of **[^18^F]1**, while HLM, HepG2 CYP2C19, and HepG2 CYP1A2 incubation produced the same metabolites. This phenomenon could be explained by the absence of CYP2C19 in mice [40] It is concluded that the cell lines reflect the human in vivo conditions comparably to HLMs and, hence, more precisely than MLMs.

Previously published data already identified hydroxylated metabolites after an incubation with liver microsomes [28]. Accordingly, **M1** originates from hydroxylation at the methylphenyl group, which was confirmed in the present study based on retention time and mass-to-charge ratio. **M2** was only observable in the present study after an incubation with CYP1A2- and CYP2C19-overexpressing HepG2 cell lines and HLM, not in the previous MLM study. After a carrier-added incubation for 25 h to allow for sufficient **M2** formation, the compound was successfully detected with LC–MS (see Supplementary Materials, Figures S9–S11). On the basis of the observed mass and daughter spectra, it can be assumed to be a carboxylic acid-bearing metabolite. Hence, it is proposed that the hydroxy group of first metabolite (**M1**) is further oxidized to a carboxyl group (Figure 1B).

### 3.3. Cell-Based Radiotracer Metabolism of [^18^F]1, [^18^F]2, and [^18^F]3

**Figure 4:**
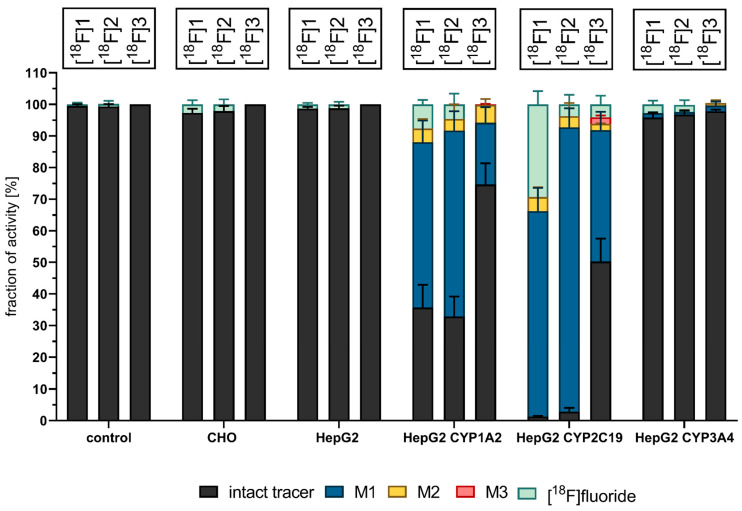
Activity distribution within supernatant after 1 h incubation with ^18^F-labeled coxib tracers (2–4 MBq/mL corresponding to a concentration range of 28–187 nM) in respective cell lines. The intact tracer (black) gets metabolized to hydroxylated metabolite M1 (blue) and carboxylated metabolite M2 (yellow) under defluorination (mint) when incubated with HepG2 CYP1A2 and CYP2C19. Radioactive compounds separated with radio-TLC and visualized with radioluminographic imaging plates. Data shown are mean values (±SD) with *n* = 4.

**Figure 5:**
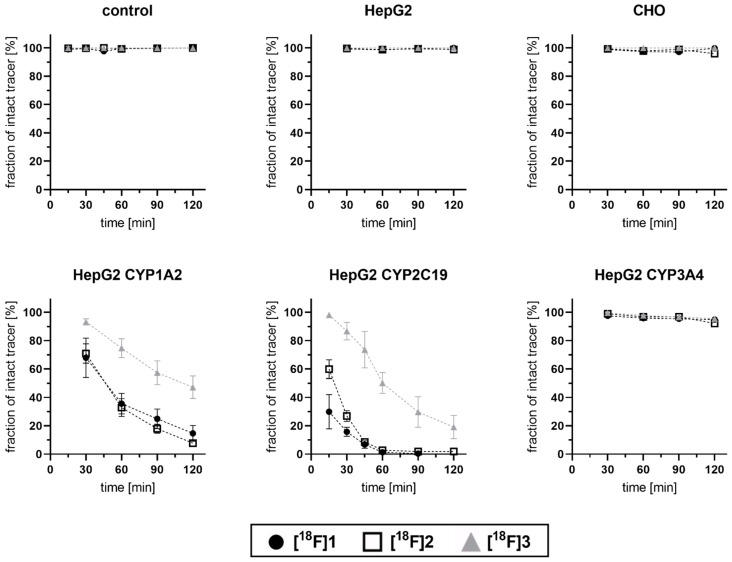
Time-dependent degradation of ^18^F-labeled coxibs in CYP-overexpressing HepG2 cell lines (2–4 MBq/mL corresponding to a concentration range of 28–187 nM). Plot of change in recorded radio-TLC signals originating from respective intact tracer in supernatant of indicated cell lines after 30, 60, 90, and 120 min. For HepG2 CYP2C19 additional timepoints at 15 min and 45 min were analyzed. Data shown are mean values (±SD) with *n* = 4.

The optimized protocol was utilized for the investigations of all three coxib radiotracers **[^18^F]1**, **[^18^F]2**, and **[^18^F]3** in three CYP-overexpressing HepG2, parental HepG2, and CHO cell lines, as well as control incubation (without any cell lines). This revealed that all three tracers were majorly metabolized by HepG2 CYP1A2 and HepG2 CYP2C19 (Figure 4 and Figure 5).

Within 1 h of incubation in HepG2 CYP2C19 with **[^18^F]1,** only 1.3% intact tracer was detected in the supernatant. The second most efficient turnover was observed by HepG2 CYP1A2, with a depletion of intact tracer **[^18^F]1** within 2 h down to 14.6%. Both cell line incubations resulted in the detection of at least one more hydrophilic metabolite. While, for **[^18^F]1** and its deuterated analog **[^18^F]2**, the formation of a main metabolite (**M1**) and one minor metabolite (**M2**) was observed, for **[^18^F]3,** one major (**M1**) and two minor metabolites (**M2** and **M3**) were detected. These most likely represent hydroxylated (**M1** and **M3**) and carboxyl-substituted (**M2**) metabolites (For a more detailed discussion on structural elucidation in corresponding nonradioactive reference incubations, see Supplementary Materials, Figures S9–S11). On the other hand, all three radiotracers were principally stable during incubations with CHO, HepG2 wildtype, and HepG2 CYP3A4. The results obtained after incubation with CHO and HepG2 wildtype are in accordance with the results derived from control incubations (without any cell line), demonstrating that CHO and HepG2 wildtype serve as suitable negative control cell lines. In these cell lines, only two radioactive species could be detected by radio-TLC, the intact tracer and [^18^F]fluoride (<5%). During incubation with **[^18^F]3** in HepG2 CYP3A4, small amounts of metabolites were detected, but in such low amounts that the summarized percentage of metabolites remained below 3%. According to the observation for the reference incubation without any cell lines, **[^18^F]1** and **[^18^F]2** seem to undergo defluorination without additional enzymes in the incubation buffer (KHB) to a minor extent. The amount of [^18^F]fluoride after a 1 h incubation period increased in the order of HepG2 wt < CHO < HepG2 CYP3A4 < HepG2 CYP1A2 < HepG2 CYP2C19 for the fluoromethyl tracer **[^18^F]1** and HepG2 wt < HepG2 CYP3A4 < CHO < HepG2 CYP2C19 < HepG2 CYP1A2 for the deuterated analog **[^18^F]2**. The process of defluorination for **[^18^F]3** was only noted during incubation with HepG2 CYP2C19.

The time-dependent degradation of the intact tracers **[^18^F]1–3** was investigated after 15–120 min incubation time (Figure 5), and results were found to be in line with the observations described above for the selected 60 min incubation timepoint. In brief, while, after incubations with CHO, HepG2, and HepG2 CYP3A4, as well as in negative control incubations, the radiotracer was found to be intact, a clear time-dependent degradation of all three radiotracers was observed in HepG2 CYP1A2 and HepG2 CYP2C19 cells. For **[^18^F]1** and its deuterated analog **[^18^F]2**, slight but inconclusive differences were observed. In HepG2 CYP1A2, a little higher stability of **[^18^F]1** was observed at later timepoints of 90 and 120 min, while, in HepG2 CYPC19, a higher stability of **[^18^F]2** was found between 15 and 30 min incubation time. On the other hand, a pronounced slower degradation of **[^18^F]3** could be detected.

**Figure 6:**
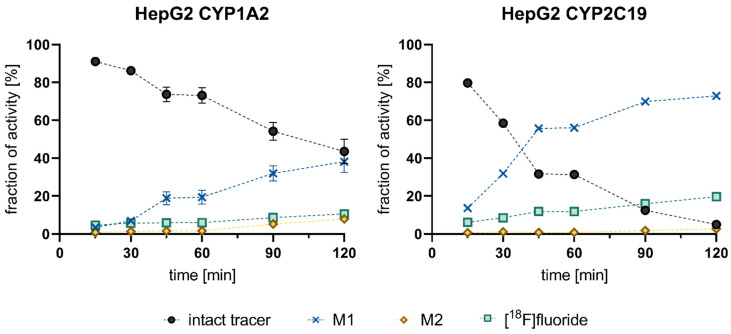
Detailed time-dependent metabolism of **[^18^F]1** (black) in HepG2 CYP1A2 (4.5 × 10^5^ cells/well) and CYP2C19 (2 × 10^5^ cells/well) to hydroxylated metabolite M1 (blue) and carboxylated metabolite M2 (yellow) under defluorination (mint). Cells were seeded into 24-well plate and cultivated for 4 days without medium change. Incubations were carried out with 1.97 MBq/well in 500 µL total volume corresponding to a concentration of 92.0 nM at the start of experiment (A_m_ = 72 GBq/µmol). The assignment of percentage activity amounts to intact tracer (R_f_ = 0.25) in this and following experiments was performed on the basis of a chromatographic comparison to **[^18^F]1** in the same chromatographic system. Metabolite 1 (R_f_ = 0.49) and metabolite 2 (R_f_ = 0.57) were the more formed hydrophilic metabolites, while [^18^F]fluoride was detected with R_f_ = 0.07. Data shown are mean values (±SD) with n = 2.

The time-dependent composition of incubation mixtures with **[^18^F]1** considering intact **[^18^F]1**, the metabolites **M1** and **M2**, and [^18^F]fluoride is shown in Figure 6. Up to 60 min, **[^18^F]1** was mainly metabolized to **M1** accompanied by simultaneous radiodefluorination. With ongoing depletion of intact tracer and formation of **M1** by HepG2 CYP1A2 and CYP2C19, the occurrence of a second metabolite (**M2**) increased markedly after 90 min, likely corresponding to the respective carboxylic acid derivative formed from **M1**. After a total incubation time of 120 min and advanced degradation of the intact tracer, the metabolite **M2** was formed in considerable amounts in the incubation supernatant of HepG2 CYP1A2 (7.9%) and HepG2 CYP2C19 (2.6%), suggesting that CYP1A2 facilitated the formation of **M2** more than CYP2C19. However, this assumption needs to be validated with additional assays utilizing isolated enzymes, since no information about protein expression levels in the utilized cell lines was generated which would allow a quantitative comparison.

The lead compound celecoxib is majorly metabolized by CYP2C9 [29,41], which belongs to the same subfamily (2C) as CYP2C19, with both isoenzymes sharing over 80% [42] amino-acid sequence identity. Overlapping substrate specificity among the members of the CYP2C subfamily can be seen in metabolic pathways of diclofenac and warfarin, for example [42]. Accordingly, the increased metabolic activity of HepG2 CYP2C19 compared to the two other CYP overexpressing cell lines can be explained.

In our previous work, the in vitro stability of **[^18^F]1**, **[^18^F]2**, and **[^18^F]3** was investigated with murine liver microsomes (MLMs) [28]. The resulting data elucidated an increase in the formation of radiometabolites in the order **[^18^F]2** < **[^18^F]1** < **[^18^F]3**. Consulting the here-described cell-based data, the opposite trend in metabolite formation can be observed. Upon incubation with HepG2 CYP1A2, the metabolite formation increased in the order of **[^18^F]3** < **[^18^F]1** < **[^18^F]2,** and, when incubated with HepG2 CYP2C19, it increased in the order of **[^18^F]3** < **[^18^F]2** < **[^18^F]1**. Both **[^18^F]1** and **[^18^F]2** tend to show similar metabolite formation when incubated in the same cell line (Figure 4). The stability of **[^18^F]1** in terms of radiodefluorination was improved by both deuteration (**2**) and elongation (**3**), but most efficiently by elongation of the alkyl chain where ^18^F is attached. In vivo experiments also revealed that the bone accumulation of ^18^F-activity remained lowest for the ethyl derivative [28]. Hence, the overall stability in CYP-overexpressing HepG2 cell lines toward the metabolic degradation and defluorination was strongest for **[^18^F]3**—the fluoroethyl-derivative of celecoxib.

### 3.4. Competitive Incubations

**Figure 7:**
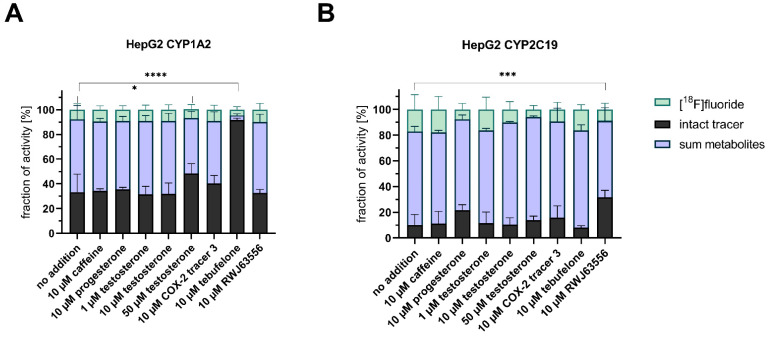
Coincubation of **[^18^F]1** and different competitors with (**A**) HepG2 CYP1A2 and (**B**) HepG2 CYP2C19. The intact tracer (black) gets metabolized to hydroxylated metabolite M1 and carboxylated metabolite M2 (depicted in a summarized manner in light blue) under defluorination (mint). Upon coincubation, the metabolite profile can be altered. Each competitor/inhibitor was incubated with **[^18^F]1** for 1 h followed by radio-TLC analysis. Activity concentrations of 94–197 kBq/well (1.9–3.9 MBq/mL) were used at start of experiment, which correspond to a concentration of 120–125 nM. Statistical analysis performed with Dunnett’s multiple comparison (post hoc test) according to the intact tracer amount (* *p* < 0.05; *** *p* < 0.001; **** *p* < 0.0001; *n* = 4).

Our last interest within this study was to test if the tracer itself could function as an indicator for the conversion of other substrates acting via inhibition of or competition with the CYP enzymes. In this regard, alternative substrates were added to the incubation mixtures with **[^18^F]1**. The investigated substrates covered testosterone, caffeine, progesterone, compound **3,** tebufelone [43], and RWJ63556 [44] (10 µM for all substances). Furthermore, the influence of testosterone on the metabolic turnover of **[^18^F]1** was investigated over a concentration range of 1–50 µM. The respective data are collectively shown in Figure 7.

Within the analyzed range of substances, only a few led to a significant increase in intact tracer, indicating an influence on the respective overexpressed CYP isoform. Coincubation in HepG2 CYP1A2 with caffeine, progesterone, testosterone, and RWJ63556 with concentrations of 1 or 10 µM resulted in no change in the metabolic profile compared to the reference incubation without any additional substance. However, a coincubation with the herein investigated tracer **3** slightly increased the intact tracer amount, and a coincubation with 50 µM testosterone, as well as coincubation with tebufelone, led to a significant increase in intact tracer **[^18^F]1** (Figure 7A). While, in the incubation without any additional substance, 33% intact tracer remained after 1 h, the amount increased by a factor of 1.5 when 50 µM testosterone was added (48%). An even more elevated increase was observed when 10 µM tebufelone was added to the incubation mixture, resulting in 2.8-fold more intact tracer (92%), which suggests an inhibitory effect on **[^18^F]1** degradation through CYP1A2. For HepG2 CYP2C19 (Figure 7B), increasing concentrations of testosterone led to a slight increase in the amount of intact radiotracer indicating a dose-dependent influence. Progesterone is described as a specific substrate of CYP2C19 [45]. The coincubation with progesterone yielded twofold more intact tracer (up to 22%) compared to the incubation without additional substances (11% intact tracer) after a 1 h incubation. The degradation of intact tracer was even more diminished in a highly significant manner by coincubation with RWJ63556 (32%, threefold increase). Of note, test compounds were found to be nontoxic for both HepG2 CYP2C19 and CYP1A2 cells when applied at the respective incubation conditions (see Supplementary Materials, Figure S14).

To summarize, this method can be used for the analysis of other, nonradioactive therapeutics toward their effects on CYP isoforms. Although competition or inhibition characteristics of different drugs cannot be delineated from these experiments alone, a changed radiotracer metabolism can serve as indicator in this setting. Because activity is easy and highly sensitive to detect, a first and basic knowledge on the specific CYP metabolism can be gained in a simplified and reliable manner. While not needed in the first place, analytical tools such as LC–MS could afterward be used to further characterize the respective interaction. This knowledge can be especially useful for patients with polymorphic CYP isoforms. The investigations with **[^18^F]1** in the presence of different competitors proved that this approach works also in a CYP isoform-dependent manner (Figure 7). While coincubations with tebufelone and the highest applied testosterone concentration of 50 µM led to a significant increase in intact tracer in HepG2 CYP1A2, no change was observed when incubated with HepG2 CYP2C19. On the other hand, RWJ63556 led to a significant increase in **[^18^F]1** in HepG2 CYP2C19, but not when incubated with HepG2 CYP1A2. To further elucidate the different influences of certain substances on CYP isoforms, an investigation in a concentration-dependent approach should be included in future experiments, as initial increases in intact tracer with increasing testosterone concentrations were observed for both CYP1A2- and CYP2C19-overexpressing HepG2. In terms of nearly complete inhibition of the degradation, a concentration-dependent investigation with ketoconazole and the addition of more selective inhibitors such as furafylline [46] for CYP1A2 or nootkatone [47,48] for CYP2C19 could serve as positive controls in future studies. 

## 4. Conclusions

The study successfully showed the suitability of CYP-overexpressing HepG2 cells for elucidating the metabolism of radiotracers bearing short-lived radionuclides. The 96-well plate format in combination with radio-TLC allowed comparably moderate to high throughput and easy sample handling and analysis, while maintaining cell viability, CYP functionality, and metabolite formation. The limitations of the described procedure mostly arise from the analytical setup, as only a limited number of samples fit onto one TLC plate, which has to be developed and read out.

For the three investigated coxib tracers, clear differences regarding CYP isoform specificity were observed. The above-presented results prove the presence of metabolites after incubation with HepG2 CYP1A2 and HepG2 CYP2C19 in radioactive experiments, as well as respective nonradioactive reference experiments. All three tracer compounds were mostly stable during incubations with CHO, HepG2 wildtype, and HepG2 CYP3A4.

The optimized protocol for the investigation of radiotracer metabolism, hence, delivers information about CYP-specific metabolism. Furthermore, it allows the investigation of the time-dependent degradation of an ^18^F-labeled radiotracer in five different cell lines within 1 day using only 30 MBq of the radiotracer. Therefore, CYP-overexpressing HepG2 cells provide a good complement to evaluate radiotracer stability and to gain insight into the CYP-specific metabolism of radiotracers. Exemplarily, the CYP2C19 isoenzyme is highly polymorphic with more than 25 known variant alleles [49,50]. Patients with such a polymorphism could be categorized as poor or rapid metabolizers depending on the genotype, with the result of a hampered or even increased tracer degradation, which might impact the outcome of a PET study. The data presented also suggest that appropriate radiotracer coincubations can be used to study the effects of multiple drugs in terms of turnover, activation, or inhibition on specific CYP isoforms. From a radiopharmaceutical point of view, insights are also gained into possibilities of targeted inhibition of selected CYP isoforms with the aim of delaying the metabolization of a radiotracer, increasing its accumulation in the target tissue, and thus, significantly improving imaging diagnostics, for example, of tumors.

## Figures and Tables

**Table 1:** Activity distribution within different incubation fractions. An activity concentration of 25 MBq/mL (1.26 MBq/well) was used at start of experiment (t_0_). The control incubation was carried out without cell monolayer.

		Control	CHO	HepG2	CYP1A2	CYP2C19	CYP3A4
Supernatant	MBq/well at t_0_	1.11	0.95	0.91	1.10	1.14	0.81
	(%)	87.8	74.8	71.7	86.9	90.7	64.2
Wash	MBq/well at t_0_	0.15	0.16	0.18	0.11	0.11	0.23
	(%)	11.7	12.8	14.4	8.9	8.9	18.4
Cell lysate	MBq/well at t_0_	-	0.09	0.14	0.03	0.02	0.20
	(%)	-	7.4	10.8	2.5	1.6	15.7

## Data Availability

The data presented in this study are contained within the article.

## Supplementary Materials

The following supporting information can be downloaded at https://www.mdpi.com/article/10.3390/cells11152447/s1: Figure S1. Radio-TLC of supernatant and cell lysates after incubation with **[^18^F]1**; Figures S2–S5. Data from method development with **[^18^F]1**; Figure S6. Data from method development using testosterone; Figure S7. UPLC–MS/MS results from time-dependent degradation of reference compounds **1**–**3**; Figures S8–S11. UPLC–MS/MS analyses of metabolites from compounds **1**–**3** for structural elucidation; Figure S12. Representative radio-HPLC chromatograms of [^18^F]**1**–**3** at the end of synthesis; Figures S13–S14. Cell growth and vitality of CYP-overexpressing cell lines. References mentioned in SM: [34] as in reference list below.
