# Supplementary material for: Investigation of Radiotracer Metabolic Stability In Vitro with CYP-Overexpressing Hepatoma Cell Lines
Source: Cells. 2022 Aug 7;11(15):2447. doi: 10.3390/cells11152447 (PMC9368227; doi:10.3390/cells11152447)
Supplement: Supplementary file 1 [file cells-11-02447-s001.zip › cells-1747694-supplementary.pdf]

cells

Supplementary Materials

Investigation of radiotracer metabolic stability *in vitro* with CYP overexpressing hepatoma cell lines

Sandy Lemm [1,2], Susanne Köhler [3], Robert Wodtke [1], Friedrich Jung [3], Jan-Heiner Küpper [3], Jens Pietzsch [1,2,#] and Markus Laube [1,#,*]

1 Helmholtz-Zentrum Dresden-Rossendorf, Institute of Radiopharmaceutical Cancer Research, Bautzner Landstrasse 400, 01328 Dresden, Germany
2 Faculty of Chemistry and Food Chemistry, School of Science, Technische Universität Dresden, Mommsenstrasse 4, D-01062 Dresden, Germany
3 Faculty of Science, Brandenburg University of Technology Cottbus-Senftenberg, Germany
J.P. and M.L. share senior authorship.
* Correspondence: m.laube@hzdr.de

Table of contents

1. Radio-TLC of supernatant and cell lysates after incubation with [^{18}F]1 2

2. Data from method development with [^{18}F]1 3

3. Data from method development using testosterone 5

4. UPLC-MS/MS results from time dependent degradation of reference compounds 1-3 6

5. UPLC-MS/MS analyses of metabolites from compounds 1-3 for structural elucidation 7

6. Representative radio-HPLC chromatograms of [^{18}F]1-3 at the end of synthesis 11

7. Cell Growth of CYP-Overexpressing cell lines 12

1. Radio-TLC of supernatant and cell lysates after incubation with [18F]1

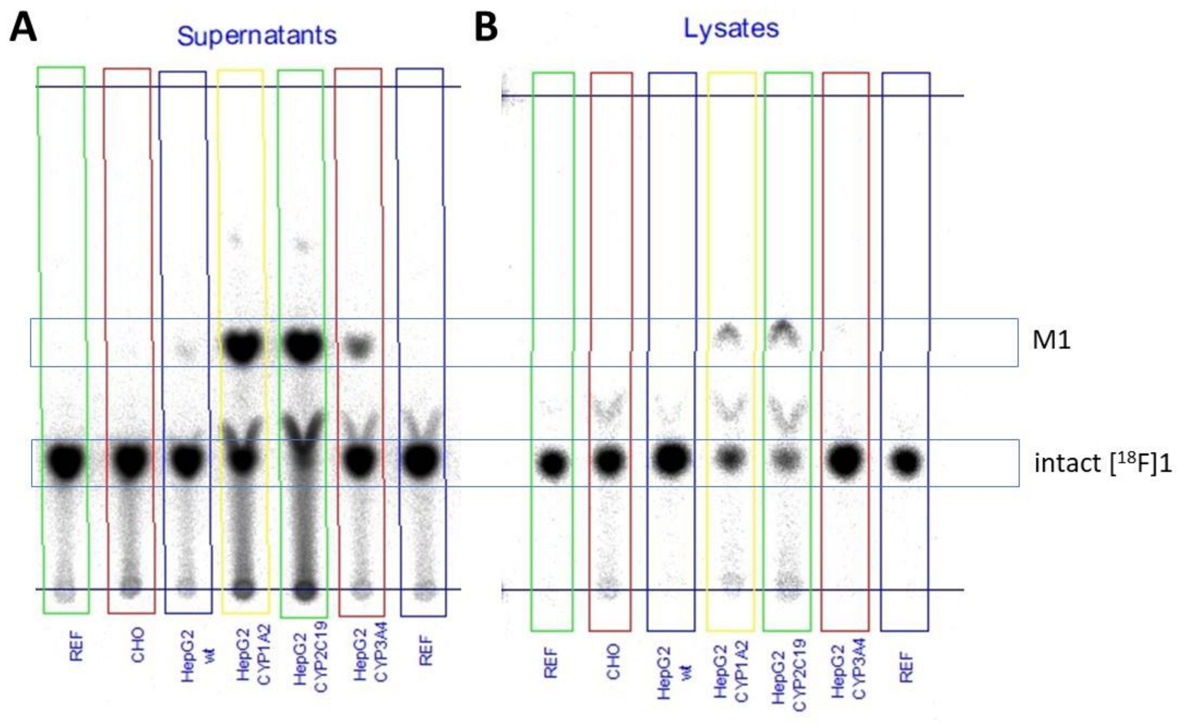

Figure S1: Radio-TLC scan after 1h incubation with [18F]1. **A:** supernatants **B:** cell lysates

2. Data from method development with [18F]1

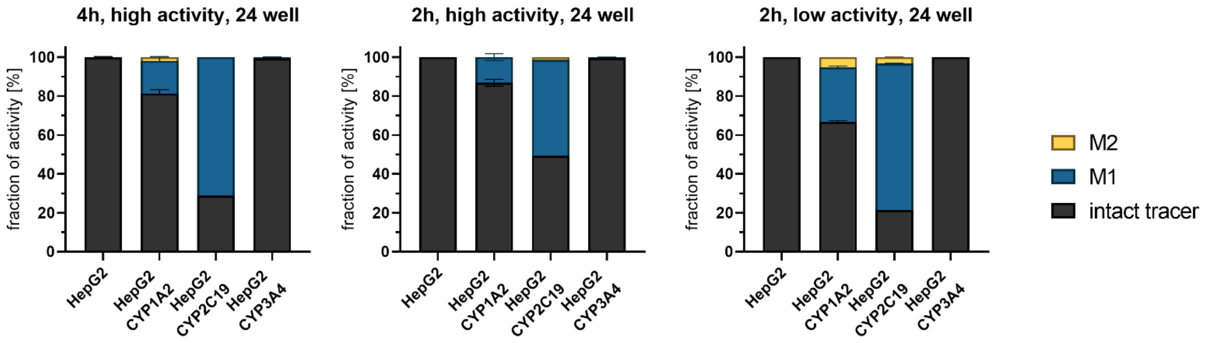

Figure S2: Comparison of incubation time and activity. Results from radio-TLC of precipitated supernatants after 2 h and 4 h incubation with [18F]**1** in CYP overexpressing HepG2. Assignment of percentual activity amounts to intact tracer (R_f = 0.25) in this and following experiments was performed based on chromatographic comparison to [18F]**1** in the same chromatographic system. Metabolite 1 (R_f = 0.49) and metabolite 2 (R_f = 0.57) resemble the formed more hydrophilic metabolites. Experiment conducted in a 24 well plate with 7.86 MBq/well (high activity concentration) corresponding to a concentration of 2.5 µM and 762 kBq/well (low activity concentration) corresponding to 220 nM at the start of experiment (0.5 % ethanol). A_m = 9 GBq/µmol; RCP < 99 %; CP = 12 %. HepG2 (P22), HepG2 CYP1A2 (P28), HepG2 CYP2C19 (P40), and HepG2 CYP3A4 (P41) in 24 well plate cultured for 3 days prior experiment. HepG2 and CYP1A2: 3.2*10^5 cells/well, CYP2C19: 1.5*10^5 cells/well, and CYP3A4: 1.3*10^5 cells/well. Data shown are mean values (±SD) with n=2.

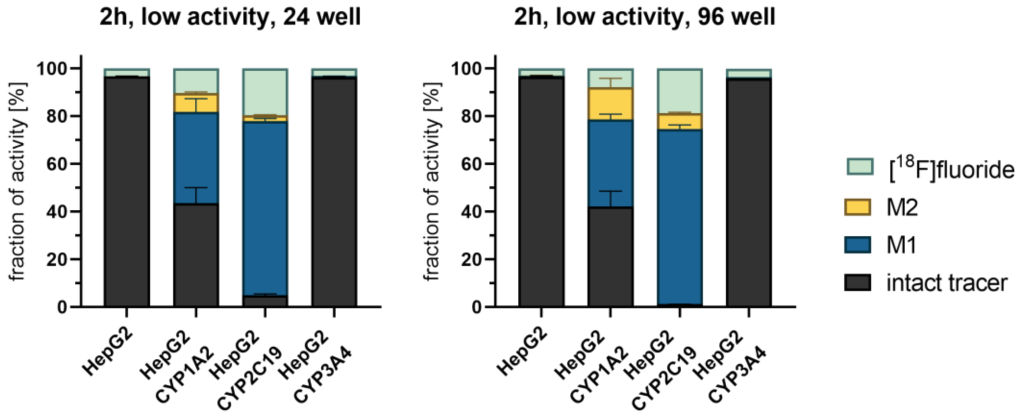

Figure S3: Comparison of well format. Results from radio-TLC of supernatants from 2 h incubations with [18F]**1** in different HepG2 cell lines. Experiment conducted in 24 well (1.97 MBq/well with 500 µL applied total volume) and 96 well plates (210 kBq/well with 50 µL applied total volume), corresponding to a concentration of 92 nM at start of experiment. A_m = 72 GBq/µmol. [18F]fluoride was detected with R_f = 0.07. HepG2 (P25), HepG2 CYP1A2 (P31), HepG2 CYP2C19 (P42), and HepG2 CYP3A4 (P43). n=2 for each cell line. Cells were seeded with different densities and cultivated for four days without media change. HepG2 seeded in 96 well plate with 8.9*10^4 and 5*10^5 cells/well in 24 well plate. HepG2 CYP1A2 seeded in 96 well plate with 7.5*$10^4$and 4.5*10^5cells/well in 24 well plate. 96 well plate HepG2 CYP2C19 seeded in with 3.97*10^4 cells/well and HepG2 CYP3A4 with 3.5*10^4 cells/well, while the 24 well plate was seeded with 2*10^5 cells/well for both cell lines. Data shown are mean values (±SD) with n=2.

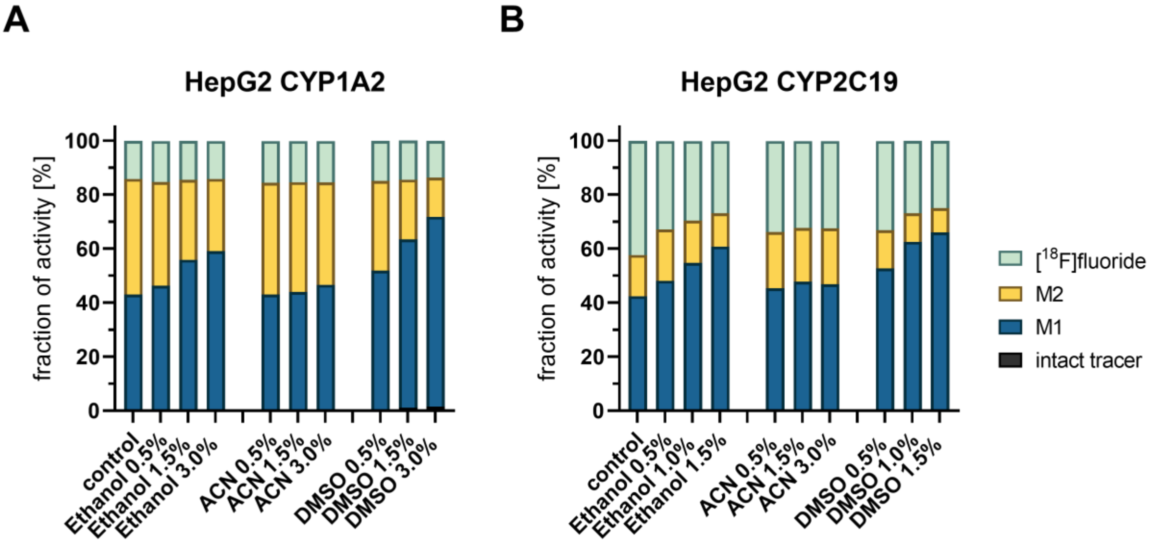

Figure S4: Influence of different solvent concentrations on [^{18}F]**1** metabolism in **(A)** HepG2 CYP1A2 and **(B)** HepG2 CYP2C19 cells. Control incubation contained 0.4 % ethanol. To the remaining incubations additional solvent concentrations (as shown on x-axis) were added. Experiment performed without replicates. Cells seeded in 96 well plate with 3*10^5 cells/well and cultured overnight. HepG2 CYP1A2 (P50) and HepG2 CYP2C19 (P35). Radioactive signals resulting from different ^{18}F-labeled compounds after 2 h incubation with [^{18}F]**1** measured with radio-TLC. 148 kBq/well (CYP1A2) and 133 kBq/well (CYP2C19) corresponding to 80.6 nM at start of experiment. A_m = 60 GBq/µmol; CP = 60 %, RCP > 99 %. n=1

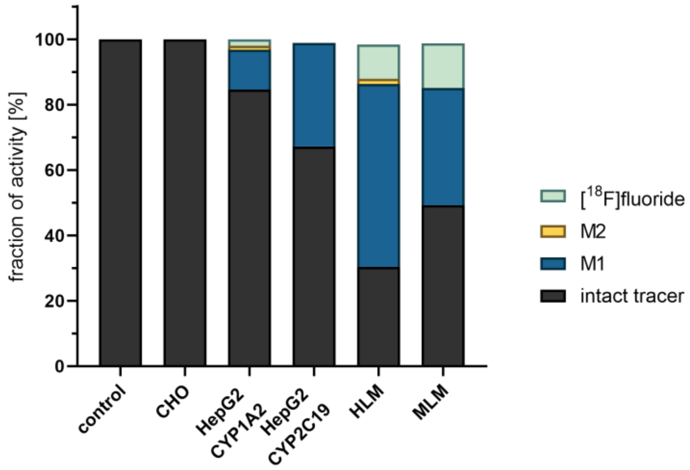

Figure S5: Comparison of cell-based and microsome-based [^{18}F]**1** metabolism under carrier-added conditions after 1h. Incubation were performed with 38 MBq/mL for microsome assays and 23.0 MBq/well for the cell-based approach and a final concentration of **1** of 50 µM. HepG2 CYP1A2 (P24), HepG2 CYP2C19 (P38), CHO (P17), MLM (MSMCP, Lot: MS046-C), HLM (HMMCPL; Lot: PL050E-B) Percental representation of activity distribution in supernatants after cell or microsome incubation as obtained from radio-TLC analysis. NC = negative control (without cells), n=1.

3. Data from method development using testosterone

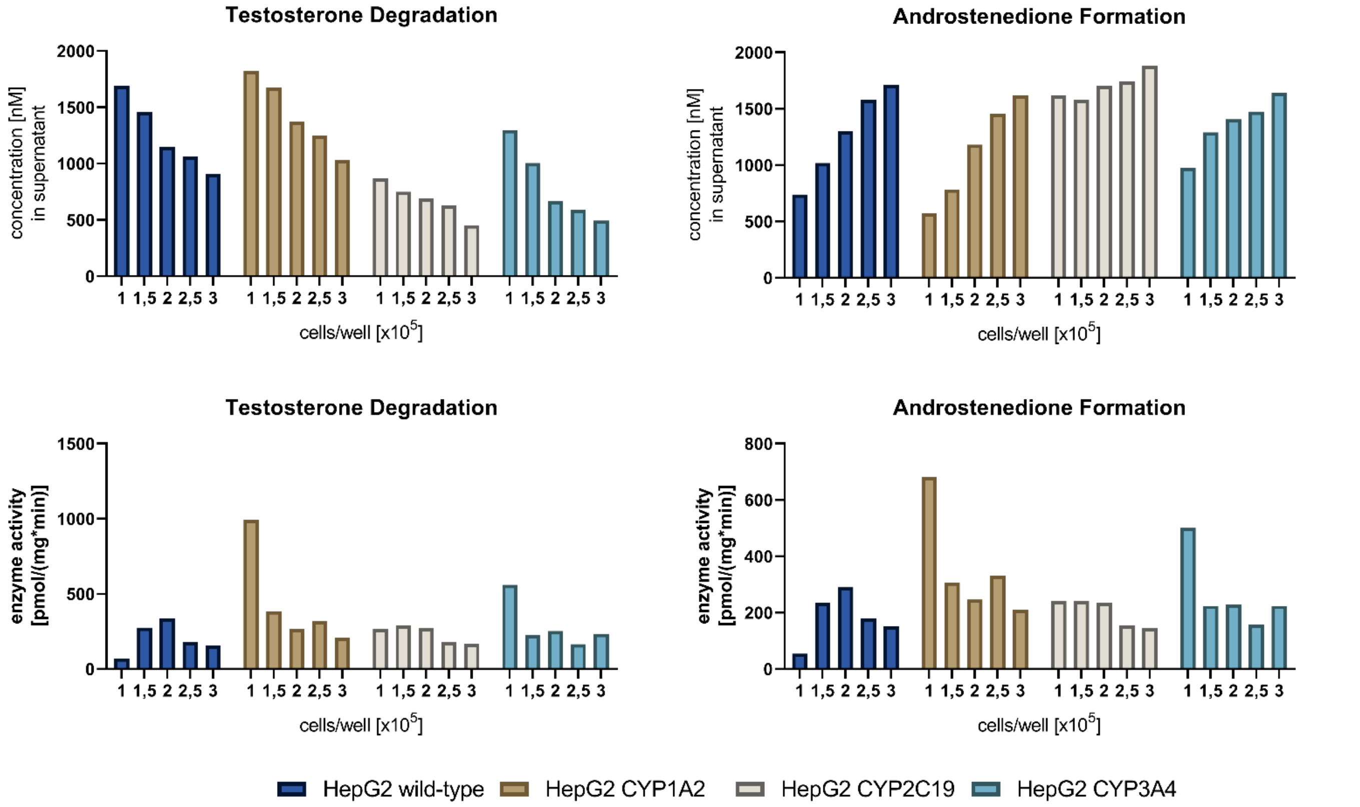

Figure S6: Influence of cell density on metabolism of 2.5 µM testosterone by different HepG2 cell lines. Upper panel: Metabolic turnover of testosterone to androstenedione measured with LC-MS/MS. Lower panel: Enzyme activity of respective cell lines based on testosterone degradation (left) and androstenedione formation (right). Cells were seeded into 96 well plate and cultured for 1 day. Incubation with total volume of 150 µL for 1,5 h. Substrate and metabolites within supernatants analyzed with UPLC-MS/MS after sample precipitation with acetonitrile. Protein amount per well determined with BCA-assay to calculated enzyme activity, n=1

4. UPLC-MS/MS results from time dependent degradation of reference compounds 1-3

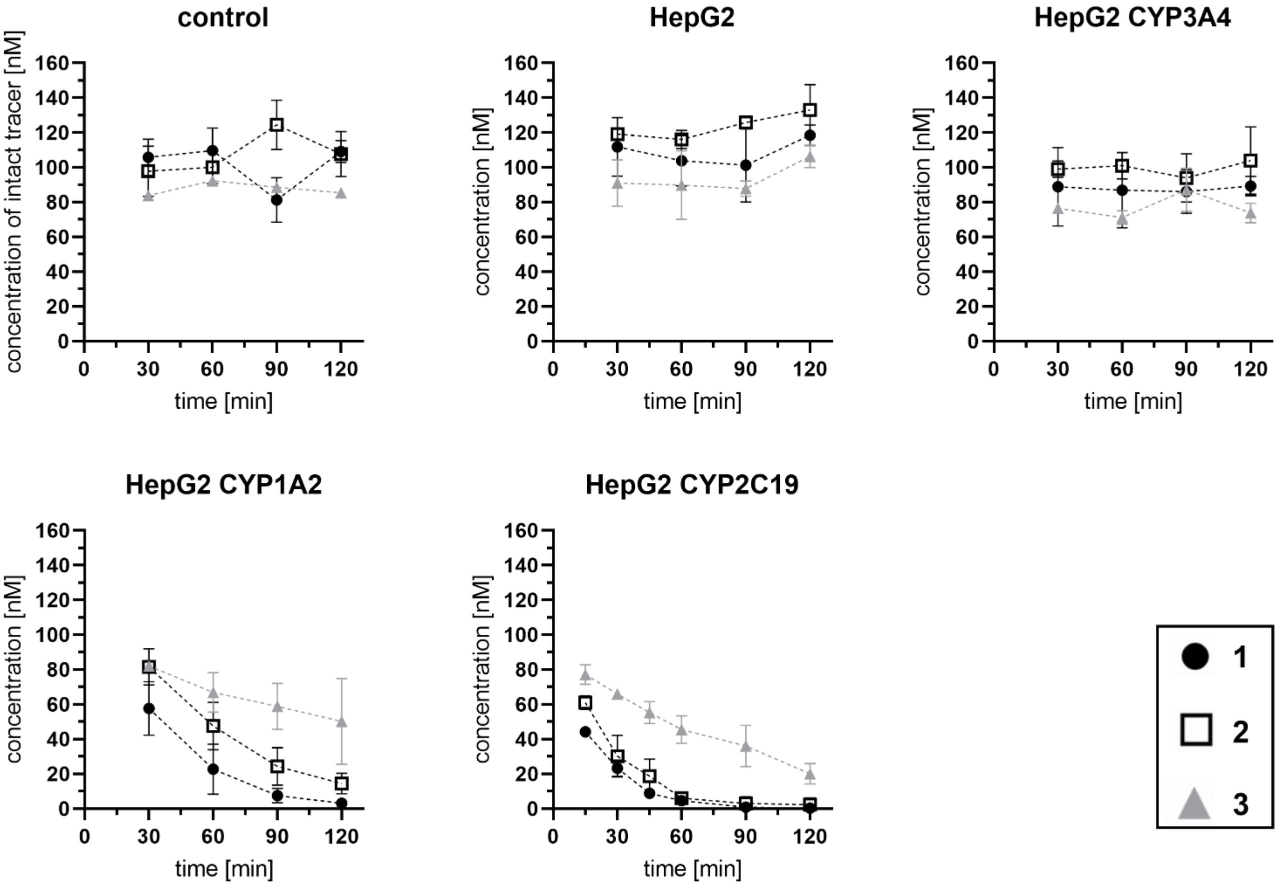

Figure S7: Time dependency of COX-2 tracer metabolism in CYP overexpressing HepG2 cell lines. Cell lines utilized: CHO (P27), HepG2 (P13, 15) HepG2 CYP1A2 (P22, 23), HepG2 CYP2C19 (P41, 42), HepG2 CYP3A4 (P37, 38), N.C. (negative control) = no cells seeded. Metabolism of 100 nM non-radioactive references substances **1-3**. Turnover of intact tracer was measured after indicated time points in precipitated supernatants by UPLC-MS/MS (system 2, 55 % isocratic elution). Data shown are mean values (±SD) with n=4 (except for control incubation with compound **3** n=1).

5. UPLC-MS/MS analyses of metabolites from compounds 1-3 for structural elucidation

As discussed in the main manuscript, for **[^{18}F]1** a direct comparison of formed metabolites in cell-based and MLM-assay (Figure 3 and in SI Figure S5) showed comparable chromatographic behavior of metabolite 1 (M1) in radio-TLC and radio-HPLC. UPLC-MS/MS measurements were performed to verify the structure of formed metabolites from **1-3**. Previously reported data with MLM [28] for carrier-added **[^{18}F]1-3** showed that hydroxylation of the tolyl-group (leading to metabolite M1) and, in case of **3**, hydroxylation at the 2-fluoroethyl-group (leading to M3) was predominant, but also defluorination to the respective alcohol occurred. In this study, also a further more hydrophilic metabolite herein named M2 was observed in small amounts after incubation with CYP1A2 and CYP2C19 overexpressing HepG2 cells and HLM. To elucidate the structure of M2, incubation of reference compounds **1-3** was performed and the metabolite profile was analyzed after 2, 5, and 24 h. Targeted UPLC-MS/MS analysis confirmed the presence of hydroxylated metabolites (M1/M3, $\Delta m/z + 16$) and a metabolite resulting from oxidation of the tolyl group to the carboxylic acid (M2, $\Delta m/z + 30$). Targeted UPLC-MS/MS daughter ion scans for the possible metabolites (Figure S9-11) are in accordance with the proposed structure of the previously identified hydroxy-bearing metabolites (M1/M3) as well as M2 herein characterized as the respective carboxylic acid derivatives.

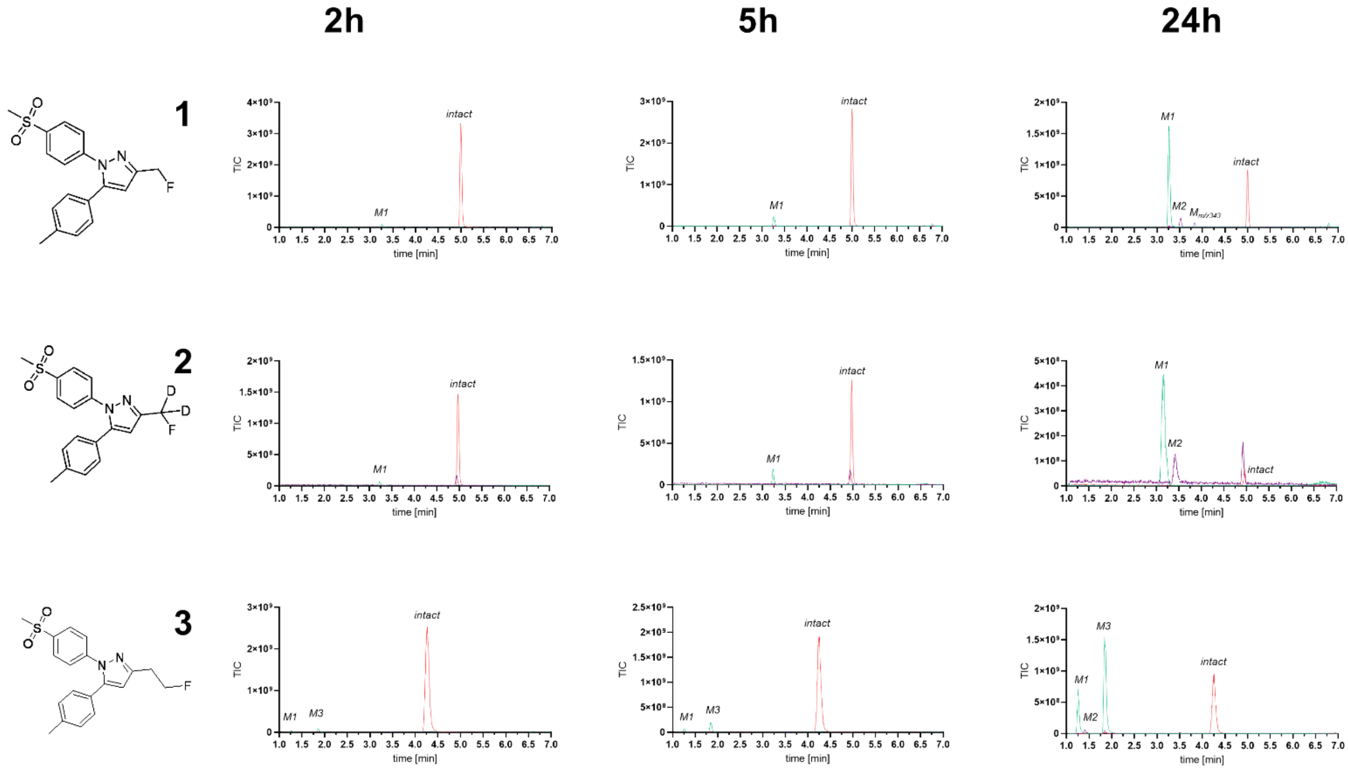

Figure S8: UPLC-MS/MS chromatograms after 2h, 5h, and 24h incubation with HepG2 CYP1A2. Chromatic separation achieved with 25-75% gradient elution for compound 1 and 2 and 50% isocratic elution for compound 3 (system 2). Signal intensity represented as total ion count (TIC).

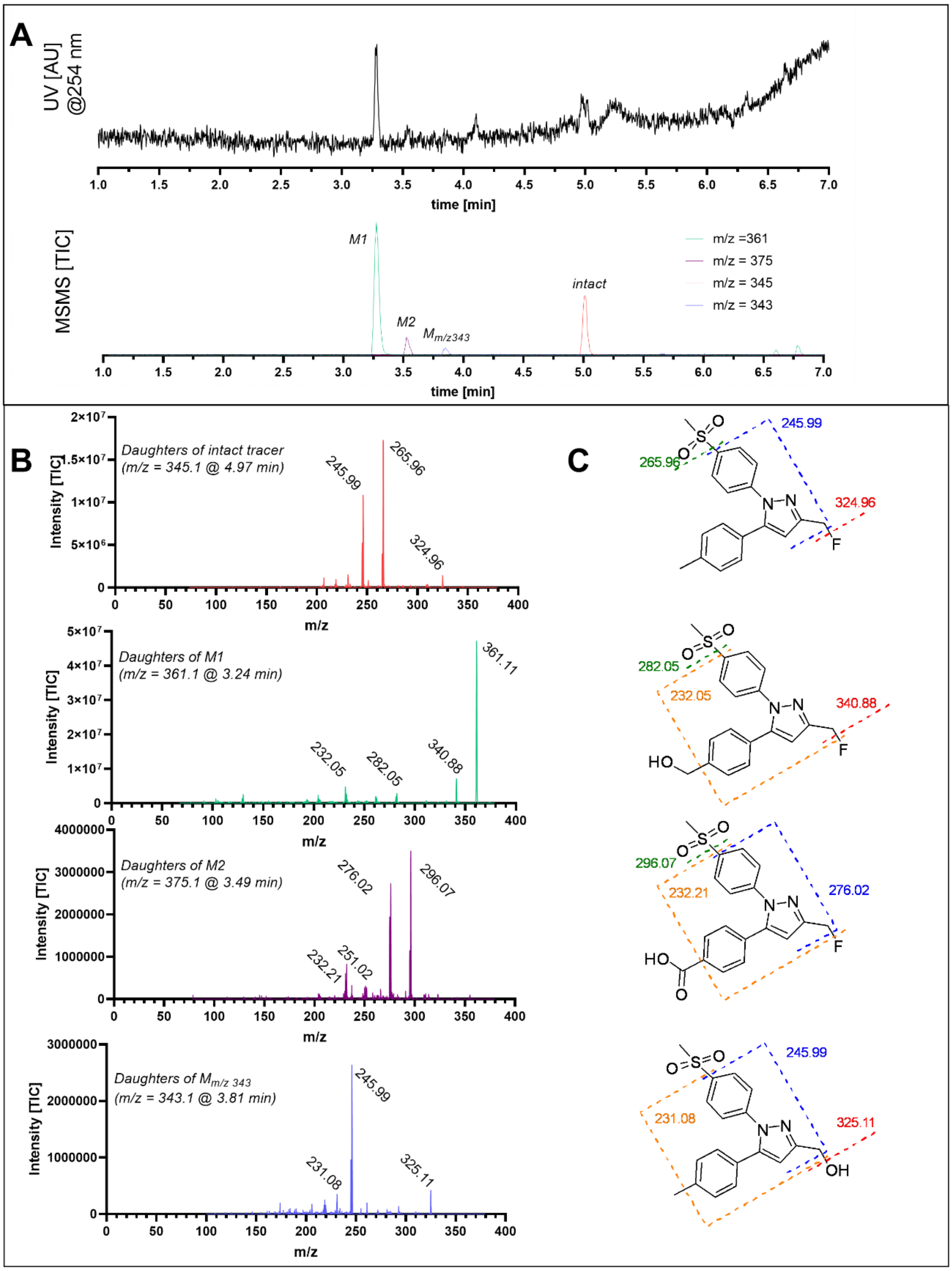

Figure S9: Chromatogram (A) and MSMS spectra (B) of tracer **1** after 25 h incubation in HepG2 CYP1A2. System 2, 25-75 % gradient elution. Corresponding fragmentation pattern (C) to spectra (B) of detected compounds labeled with measured m/z ratios

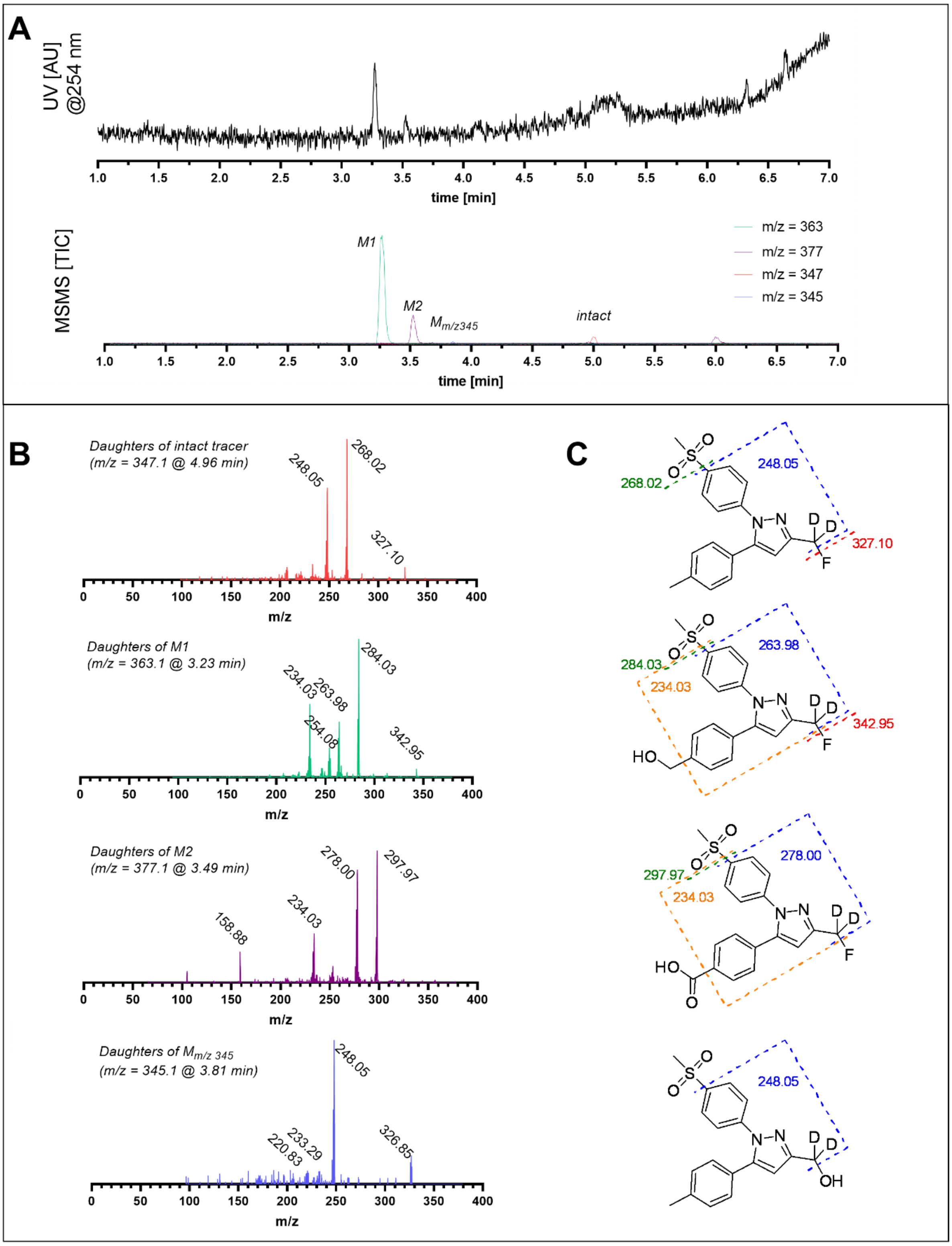

Figure S10: Chromatogram (A) and MSMS spectra (B) of tracer **2** after 25 h incubation in HepG2 CYP1A2. System 2, 25-75 % gradient elution. Corresponding fragmentation pattern (C) to spectra (B) of detected compounds labeled with measured m/z ratios

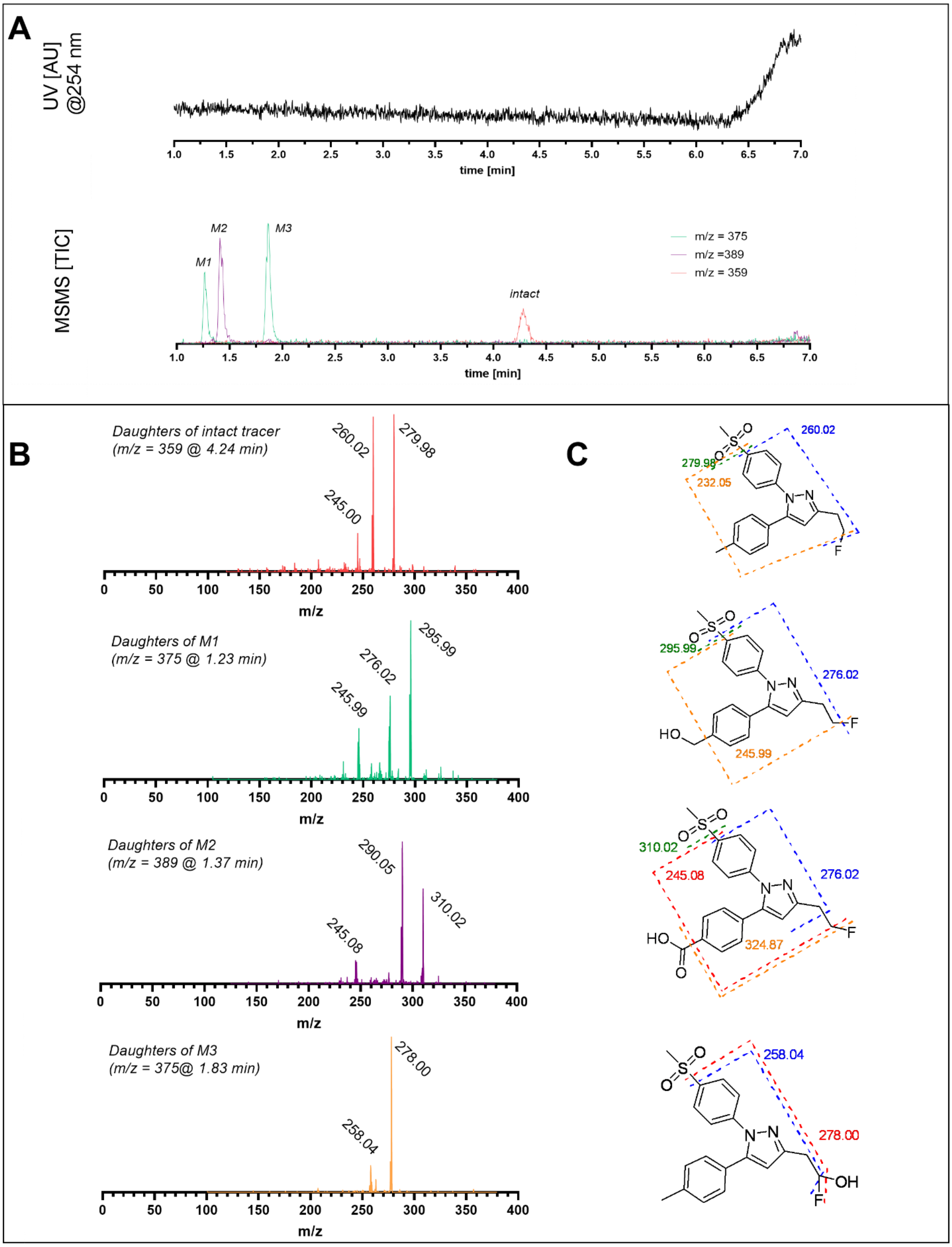

Figure S11: Chromatogram (A) and MSMS spectra (B) of tracer 3 after 25 h incubation in HepG2 CYP1A2. System 2, 50 % isocratic elution. Corresponding fragmentation pattern (C) to spectra (B) of detected compounds labeled with measured m/z ratios

6. Representative radio-HPLC chromatograms of [18F]1-3 at the end of synthesis

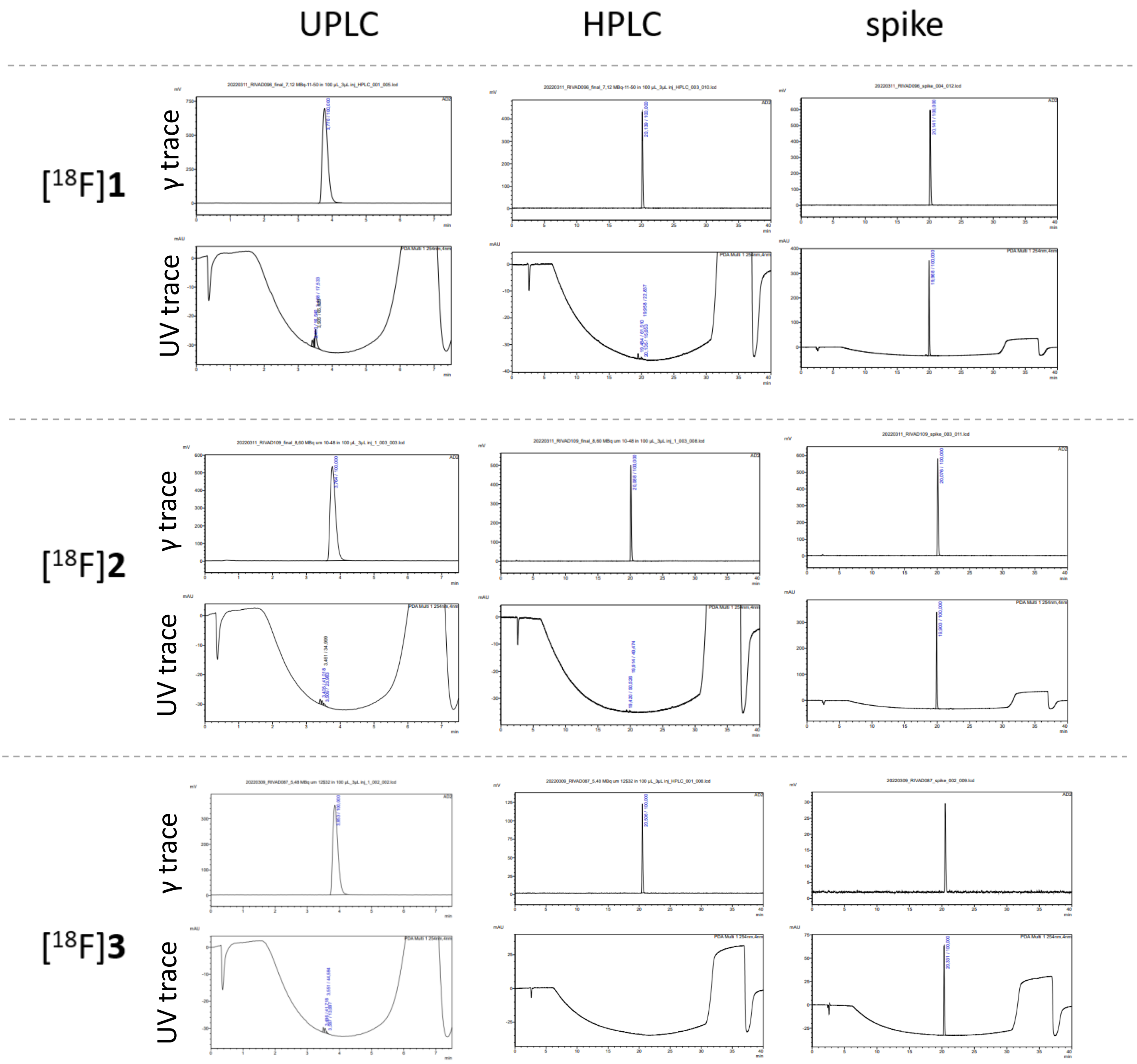

Figure S12: Analytical radio-UPLC (A) and -HPLC (B, C) after radiotracer synthesis of the final product (A, B) and in a mixture with the respective non-radioactive reference compound (C).

7. Cell Growth of CYP-Overexpressing cell lines

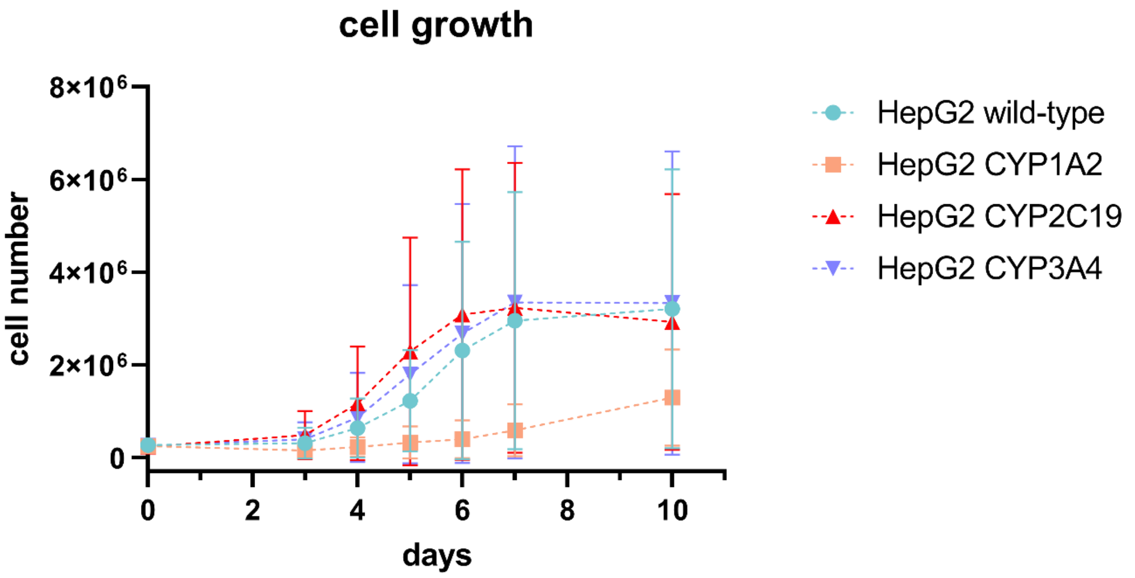

Figure S13: Growth curves of CYP-overexpressing cell lines observed over 10 days. Cell amount per well (6 well plate) was determined with CasyTon cell counter system. Data was generated using duplicates with n=3.

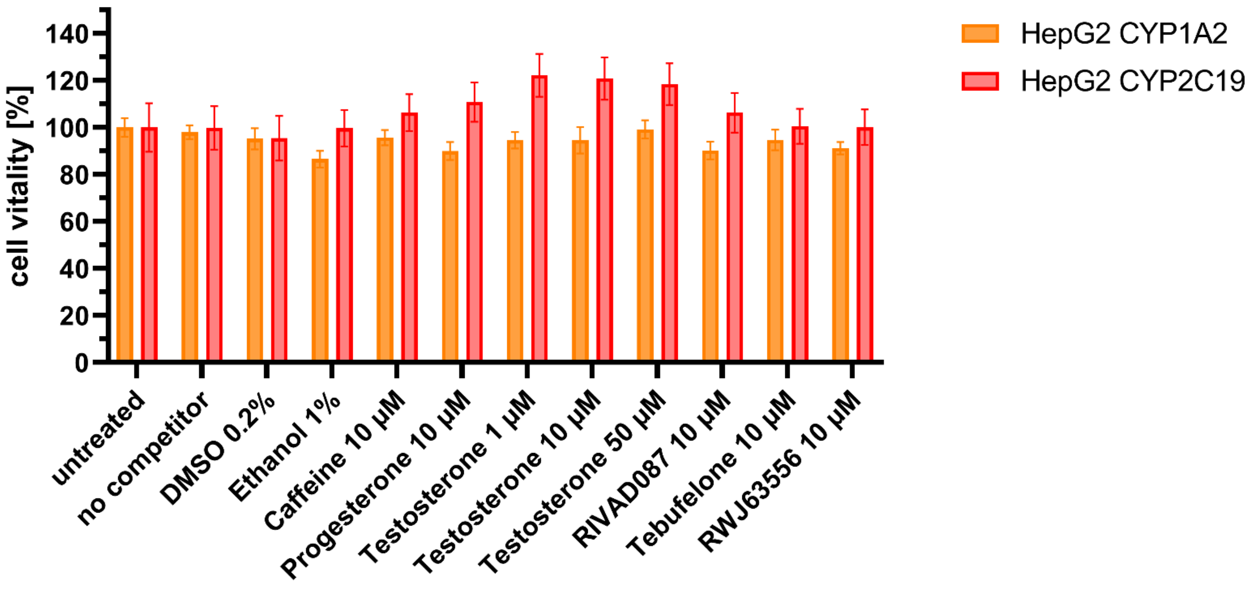

Figure S14: Cell vitality assay (MTT) according to competitor incubations in Figure 7. Cells were incubated according to incubation mixtures described in the main manuscript with indicated concentrations of competitors and controls. Cell vitality was measured with an 1h incubation of 10 µL MTT (3-(4,5-dimethylthiazol-2-yl)-2,5-diphenyltetrazolium bromide) followed by removal of supernatant and addition of 50 µL DMSO. After 10 min shaking at room photometric measurements were performed at 570 nm and 690 nm. Depiction of mean ± SEM after reference and blank subtraction, Statistical analysis was performed with Tukey's Test, n=3, but no significant deviations were found.
